# Identifying off-target effects of etomoxir reveals that carnitine palmitoyltransferase I is essential for cancer cell proliferation independent of β-oxidation

**DOI:** 10.1371/journal.pbio.2003782

**Published:** 2018-03-29

**Authors:** Cong-Hui Yao, Gao-Yuan Liu, Rencheng Wang, Sung Ho Moon, Richard W. Gross, Gary J. Patti

**Affiliations:** 1 Department of Chemistry, Washington University, St. Louis, Missouri, United States of America; 2 Department of Medicine, Washington University School of Medicine, St. Louis, Missouri, United States of America; 3 Department of Internal Medicine, Division of Bioorganic and Molecular Pharmacology, Washington University School of Medicine, St. Louis, Missouri, United States of America; Duke University, United States of America

## Abstract

It has been suggested that some cancer cells rely upon fatty acid oxidation (FAO) for energy. Here we show that when FAO was reduced approximately 90% by pharmacological inhibition of carnitine palmitoyltransferase I (CPT1) with low concentrations of etomoxir, the proliferation rate of various cancer cells was unaffected. Efforts to pharmacologically inhibit FAO more than 90% revealed that high concentrations of etomoxir (200 μM) have an off-target effect of inhibiting complex I of the electron transport chain. Surprisingly, however, when FAO was reduced further by genetic knockdown of *CPT1*, the proliferation rate of these same cells decreased nearly 2-fold and could not be restored by acetate or octanoic acid supplementation. Moreover, *CPT1* knockdowns had altered mitochondrial morphology and impaired mitochondrial coupling, whereas cells in which CPT1 had been approximately 90% inhibited by etomoxir did not. Lipidomic profiling of mitochondria isolated from *CPT1* knockdowns showed depleted concentrations of complex structural and signaling lipids. Additionally, expression of a catalytically dead *CPT1* in *CPT1* knockdowns did not restore mitochondrial coupling. Taken together, these results suggest that transport of at least some long-chain fatty acids into the mitochondria by CPT1 may be required for anabolic processes that support healthy mitochondrial function and cancer cell proliferation independent of FAO.

## Author summary

Oxidation of long-chain fatty acids inside of the mitochondrial matrix provides an essential source of energy for some cells. Since long-chain fatty acids cannot freely pass into the mitochondrial matrix, they rely on a protein called carnitine palmitoyltransferase I (CPT1) for transport. Prior research has found that many tumors exhibit increased expression of *CPT1* and/or sensitivity to CPT1 inhibition by a drug called etomoxir. These findings have led to thinking that cancer cells rely on fatty acid oxidation for energy. Here we present data that indicate otherwise, showing that inactivation of fatty acid oxidation has no effect on the proliferation of at least some cancer cell lines. Instead, these cells alter their utilization of other nutrients (such as glutamine) to compensate for the loss of fatty acid oxidation. We describe 2 discoveries that provide new insight into the role of fatty acid oxidation in cancer and help rationalize previous results. First, etomoxir has the off-target effect of inhibiting complex I of the electron transport chain. Second, CPT1 has other cellular functions that are independent of fatty acid oxidation. We suggest that one such function may be importing long-chain fatty acids into the mitochondria for anabolic fates, rather than catabolic oxidation.

## Introduction

During the last decade, carnitine palmitoyltransferase I (CPT1) has been identified as a potential therapeutic target for a growing list of cancers that include breast cancer, prostate cancer, glioblastoma, colon cancer, gastric cancer, myeloma, and others [1–6]. In these cancers, *CPT1* expression is increased, and/or CPT1 inhibition is reported to have antitumor effects. CPT1 is an enzyme associated with the outer mitochondrial membrane that transfers a long chain acyl group from coenzyme A to carnitine [7, 8]. Importantly, this transformation is required to transport long-chain fatty acids into the mitochondrial matrix. Long-chain fatty acids reaching the mitochondrial matrix are generally assumed to be oxidatively degraded, thereby implicating fatty acid oxidation (FAO) as a potentially important pathway in cancer metabolism [9].

FAO is thought to support cancer metabolism primarily in 2 ways. First, given their highly reduced state, fatty acids may provide an important source of ATP to fuel tumor growth [10]. For every pair of carbons in a fatty acid that is completely oxidized, up to 14 ATP can be produced—assuming NADH and FADH_2_ yield 2.5 and 1.5 ATP, respectively [11]. Ten of these 14 ATP are produced by oxidizing acetyl-CoA in the tricarboxylic acid (TCA) cycle. Oxidation of exogenous fatty acids might be particularly relevant to tumors that grow in adipocyte-rich environments, such as breast cancer [12]. Here, fatty acids transported from neighboring adipocytes may constitute an important energy reservoir [13]. A second potential benefit of cancer cells oxidizing fatty acids is the production of NADPH [14]. Although FAO does not make NADPH directly, the acetyl-CoA it produces in the mitochondria can be shuttled to the cytosol as citrate once acetyl-CoA condenses with oxaloacetate. Each molecule of citrate exported to the cytosol can then produce 1 molecule of NADPH through either isocitrate dehydrogenase 1 or malic enzyme 1. It has been suggested that some cancer cells rely on this source of NADPH to neutralize oxidative stress [9]. Indeed, inhibition of CPT1 in human glioblastoma cells causes a reduction in NADPH levels and an increase in reactive oxygen species [15].

A major challenge of considering FAO as an essential pathway in cancer metabolism is that cancer cells are also thought to rely heavily on fatty acid synthesis [16]. While one can rationalize the coexistence of FAO and fatty acid synthesis on the basis of subcellular compartmentalization, conventional thinking would indicate that it is unproductive to run both pathways simultaneously [9]. Additionally, recent data from our laboratory suggest that such a futile cycling process occurs to only a minimal extent in at least some proliferating cells [17].

As noted, the focus on FAO in cancer cells has mostly been driven by experimental findings related to CPT1 [6]. The assumption has been that increased *CPT1* expression and sensitivity to CPT1 inhibition represents a demand for FAO. In this work, we consider an alternative possibility that CPT1 has important metabolic roles independent of FAO. We present evidence that long-chain fatty acids transported into the mitochondria via CPT1 have important anabolic fates that are essential for proliferation. We also provide data suggesting that etomoxir, a drug commonly used to inhibit CPT1 in cancer studies, has off-target effects that may complicate the interpretation of some experiments.

We focus much of our attention on the breast cancer cell line BT549, because the essential role of CPT1 in these cells has already been thoroughly demonstrated [18]. We show that inhibiting FAO by as much as 90% had no effect on BT549 cell proliferation. At this level of pharmacological CPT1 inhibition, minimal labeling from ^13^C-enriched fatty acids could be detected in citrate. These results suggest that BT549 cells do not require FAO as a major source of ATP or NADPH. When *CPT1* is knocked down, however, we found that BT549 cell proliferation was significantly reduced. Under these conditions, the function of the mitochondria was impaired, and changes in the levels of complex lipids within mitochondria were detected. The cells could not be rescued by acetate or octanoic acid supplementation. These data support a role for CPT1 in the proliferation of some cancer cells that is independent of FAO.

## Materials and methods

### Cell culture and proliferation assays

All cells were cultured in high-glucose DMEM (Life Technologies) containing 10% FBS (Life Technologies) and 1% penicillin/streptomycin (Life Technologies) at 37 °C with 5% CO_2_. All culture media for growing cells were supplemented with 100 μM palmitate-BSA and 100 μM oleate-BSA to approach the physiological concentrations of free fatty acids. When counting cells manually, BT549 cell media were refreshed to control or experimental media 24 hours after the cells were seeded (at t = 0) to assess growth. At selected time points, cells were collected and counted in trypan blue with an automatic cell counter (Nexcelom). Doubling time was calculated by linear regression against the logarithm of cell density in exponential phase. For assessing proliferation, cells were grown under various experimental conditions for 48 hours, and proliferation was determined by using an MTT assay (ATCC) according to the manufacturer’s instructions. Absorbance was measured at 570 nm by using the Cytation 5 microplate reader (BioTek) with a reference wavelength set at 670 nm. We note that comparable changes in cell proliferation were measured using the MTT assay and manual cell counting when BT549 cells were treated with 200 μM etomoxir for 48 hours (S1 Fig), indicating that the 2 techniques to assess cell proliferation provided consistent results in our experiments. Etomoxir was purchased from Cayman Chemical (purity ≥ 98%). Etomoxir was dissolved in water to create a concentrated stock solution. The vehicle control was water alone.

### Knockdown and overexpression of *CPT1A*

*CPT1A* silencing was achieved by using a validated pool of small interfering RNA (siRNA) duplexes directed against human *CPT1A* (Trifekta Kit, IDT) and Lipofectamine RNAiMAX Transfection Reagent (Invitrogen) according to the manufacturer’s instructions (see S1 Text for the dicer-substrate short interfering RNA [DsiRNA] sequence) [19]. The knockdown (KD) efficiency was determined by measuring *CPT1A* mRNA levels with a premade primer (IDT) and quantitative RT-PCR (Applied Biosystems). The expression levels were normalized to an HPRT endogenous control. Cells given scrambled siRNA were used as a negative control. For overexpression of human *CPT1A*, the cDNA was cloned in the pcDNA3.1+ vector (GenScript) under a constitutive CMV promoter. The codon was optimized to be resistant to the siRNA added. The catalytically dead CPT1A had an identical sequence (see S1 Text) to the wild-type siRNA-resistant CPT1A, with the exception of G709E and G710E mutations to abolish catalytic activity (GenScript). For transduction, *CPT1A* was first knocked down with Lipofectamine RNAiMAX for 24 hours. Next, cells were transduced with plasmids using Lipofectamine 3000 (Invitrogen) for 4 hours. Media were then refreshed, and cells were assayed 48 hours post plasmid transduction (72 hours post siRNA knockdown). The control vector was pcDNA3.1+N-eGFP (GenScript), which expresses GFP instead of CPT1A.

### Attempts to rescue *CPT1A* knockdowns with nutrient supplementation

BT549 cells were treated with either a scrambled siRNA control or siRNA targeting CPT1A for 12 hours. Next, nutrients were added to each culture plate and incubated for 48 hours before assessing cell proliferation with an MTT assay. Each compound (sodium acetate, octanoic acid, uridine, and sodium pyruvate) was added separately and evaluated in an independent experiment relative to vehicle controls. For sodium acetate, the vehicle control was sodium chloride.

### Immunoblot analysis

Cells were lysed with RIPA buffer (Thermo Fisher Scientific) in the presence of a protease inhibitor cocktail (Thermo Fisher Scientific) and sonicated for 30 seconds. Lysates were separated by SDS-PAGE under reducing conditions, transferred to a PVDF membrane, and analyzed by immunoblotting. Rabbit anti-CPT1A (No. 12252) (Cell Signaling Technology) was used as a primary antibody. Immunoblotting for β-tubulin by mouse anti-β-tubulin antibody (Santa Cruz Biotechnology) and COX IV by rabbit anti-COX IV antibody (Cell Signaling) was used as a loading control for whole-cell lysates and mitochondrial lysates, respectively. Anti-rabbit and anti-mouse secondary antibodies were from Cell Signaling Technology and Thermo Fisher Scientific, respectively. Signal was detected using the ECL system with X-ray film development (Thermo Fisher Scientific and GE Healthcare Life Sciences) or a LI-COR C-Digit blot scanner (LI-COR) according to the manufacturer’s instructions.

### Measurement of NADH/NAD^+^ ratio

Cells were preincubated with the vehicle control or 200 μM etomoxir for 48 hours. On the day of the assay, cells were trypsinized, washed 2 times with cold PBS buffer, and extracted according to the manufacturer’s instructions. The NADH/NAD^+^ ratio was measured and calculated using an NAD/NADH Quantification Colorimetric Kit (BioVision).

### Palmitate, glucose, and glutamine labeling experiments

To assess the activity of FAO, cells were treated with vehicle control, etomoxir, scrambled siRNA, or *CPT1A* siRNA for 48–72 hours. Next, the medium was refreshed with new medium containing 100 μM uniformly ^13^C labeled (U-^13^C) palmitate-BSA and 100 μM natural abundance oleate-BSA. After labeling for 24 hours, cells were harvested, extracted, and analyzed as previously described [17]. For U-^13^C glucose, U-^13^C glutamine, and U-^13^C palmitate tracing experiments, cells were transferred to media containing ^13^C label and either vehicle control or 200 μM etomoxir for 12 hours, 6 hours, and 24 hours, respectively. The polar portion of the extract was separated by using a Luna aminopropyl column (3 μm, 150 mm × 1.0 mm I.D., Phenomenex) coupled to an Agilent 1260 capillary HPLC system. Mass spectrometry detection was carried out on an Agilent 6540 Q-TOF coupled with an ESI source operated in negative mode. Isotopic labeling was assessed comprehensively by using the X^13^CMS software [20]. The identity of each metabolite was confirmed by matching retention times and MS/MS fragmentation data to standard compounds. The isotopologue distribution patterns presented were obtained from manual evaluation of the data and calculated by normalizing the sum of all isotopologues to 100%. Data presented were corrected for natural abundance and isotope impurity.

### Nutrient-uptake analysis

After incubating cells in fresh media for 24 hours, the spent media were collected and analyzed. Known concentrations of U-^13^C internal standards (glucose, lactate, glutamine, glutamate, and palmitate; Cambridge Isotopes) were spiked into media samples before extraction. Extractions were performed in glass to avoid plastic contamination as previously reported [21]. Samples were measured by LC/MS analysis, with the method described above. For each compound, the absolute concentrations were determined by calculating the ratio between the fully unlabeled peak from samples and the fully labeled peak from standards. The consumption rates were normalized by cell growth over the experimental time period.

### Isolation of mitochondria

Mitochondria were isolated from BT549 cells as previously described [22]. In brief, cells were harvested, pelleted, and resuspended in cold mitochondrial isolation medium (MIM) (300 mM sucrose, 10 mM sodium 4-(2-hydroxyethyl)-1-piperazineethanesulfonic acid [HEPES], 0.2 mM ethylenediaminetetraacetic acid [EDTA], and 1 mg/mL bovine serum albumin [BSA], pH 7.4). Cells were then homogenized with a glass-Teflon potter. After homogenization, samples were centrifuged at 700 g at 4 °C for 7 minutes. The supernatant containing mitochondria was centrifuged again at 10,000 g for 10 minutes. Mitochondrial pellets were washed with cold BSA-free MIM, and the protein amount was determined by using a Bradford protein assay (Bio-Rad).

### Oxygen consumption assays

The oxygen consumption rate (OCR) of whole cells and isolated mitochondria was determined by using the Seahorse XFp Extracellular Flux Analyzer (Seahorse Bioscience). Cells were first incubated with vehicle control, 10 μM etomoxir, or 200 μM etomoxir for 1 hour prior to measuring respiration (we note that etomoxir was present in the assay medium as well). For *CPT1A* knockdowns, cells were treated with scrambled siRNA control or *CPT1A* siRNA for 48 hours. Cells were trypsinized and plated on a miniplate with the same seeding density 24 hours prior to the Seahorse assay. The assay medium consisted of 25 mM glucose, 4 mM glutamine, 100 μM palmitate-BSA, and 100 μM oleate-BSA in Seahorse base medium. The OCR was monitored upon serial injections of oligomycin (oligo, 2 μM), FCCP (1 μM, optimized), and a rotenone/antimycin A mixture (rot/AA, 1 μM). To measure the respiration of isolated mitochondria, freshly isolated mitochondria from BT549 cells were resuspended in cold mitochondrial assay solution (MAS). For the composition of MAS, see [22]. Samples were loaded on a miniplate with 5 μg of protein per well. Mitochondria were attached to the plate by centrifuging at 2,000 g (4 °C) for 20 minutes. After centrifugation, prewarmed MAS-containing substrates (10 mM pyruvate, 2 mM malate, 4 mM adenosine diphosphate (ADP), vehicle control, or etomoxir) were added to each well without disturbing the mitochondrial layer and then inserted into the XFp analyzer. OCR was monitored upon serial injections of rotenone (rot, 2 μM), succinate (suc,10 mM), and antimycin A (AA, 4 μM). Whole-cell OCR was normalized to the final cell number as determined by manual cell counting. Data presented were corrected for nonmitochondrial respiration.

### Confocal fluorescence microscopy

Cells were incubated with 100 nM MitoTracker Red CMXRos (Thermo Fisher Scientific) or 4 μM JC-1 (Cayman Chemical) dissolved in complete media at 37 °C for 30 minutes. Cells were washed twice with PBS and then subjected to live imaging, or cells were fixed with 4% paraformaldehyde in PBS. Fixed cells were permeabilized with 0.1% Triton X-100 (Sigma Aldrich). Next, cells were washed twice with PBS, and nuclei were stained with DAPI. Cells were then mounted with ProLong Gold (Thermo Fisher Scientific). For live imaging, nuclei were stained with Hoechst 33342 (Thermo Fisher Scientific). Cells were imaged using a Zeiss LSM 880 confocal microscope equipped with Airyscan. Images were acquired with a Zeiss 20x, 40x, 63x/1.4 NA objective using the ZEN Black acquisition software. Samples were excited with 405 (for DAPI and Hoechst 33342), 514 (for JC-1 monomers), and 543 (for Mitotracker Red and JC-1 aggregates) laser lines. Images were processed and prepared using the ZEN Black software.

### Transmission electron microscopy

Samples were fixed in 2% paraformaldehyde/2.5% glutaraldehyde (Polysciences) in 100 mM sodium cacodylate buffer, pH 7.2, for 1 hour at room temperature. Samples were washed in sodium cacodylate buffer and postfixed in 1% osmium tetroxide (Polysciences) for 1 hour. Next, samples were rinsed extensively in dH_2_O prior to en bloc staining with 1% aqueous uranyl acetate (Ted Pella) for 1 hour. Following several rinses in dH_2_O, samples were dehydrated in a graded series of ethanol and embedded in Eponate 12 resin (Ted Pella). Sections of 95 nm were cut with a Leica Ultracut UCT ultramicrotome (Leica Microsystems), stained with uranyl acetate and lead citrate, and viewed on a JEOL 1200 EX transmission electron microscope (JEOL USA) equipped with an AMT 8 megapixel digital camera and AMT Image Capture Engine V602 software (Advanced Microscopy Techniques).

### Lipidomic analysis

Isolated mitochondria (with known concentrations of internal standards) were extracted with chloroform/methanol/water (1:1:1) and vortexed for 1 minute. After centrifuging at 3,000 g for 15 minutes, the chloroform layer was dried under nitrogen gas and reconstituted with methanol/chloroform (95:5) according to the protein amount. Samples were separated using a Kinetex evo C18 column (2.6 um, 150 mm × 2.0 mm I.D., Phenomenex) coupled to an Agilent 1290 UPLC system. Mass spectrometry detection was carried out on an Agilent 6540 Q-TOF or a Thermo Scientific Q Exactive Plus coupled with an ESI source operated in both negative mode and positive mode. The lipid identities were confirmed by accurate mass as well as by matching retention times and MS/MS fragmentation patterns to standards. Absolute quantitation was achieved by normalizing to internal standards for (PC(14:1/14:1), PE(16:1/16:1), CL(14:0/14:0/14:0/14:0), PG(15:0/15:0), PS(14:0/14:0), PA(12:0/12:0), LPE(14:0), LPC(17:0), SM(d18:1/12:0), and Cer(d18:1/17:0)).

## Results

### Pharmacologically inhibiting approximately 90% of FAO does not affect cell proliferation

**Fig 1:**
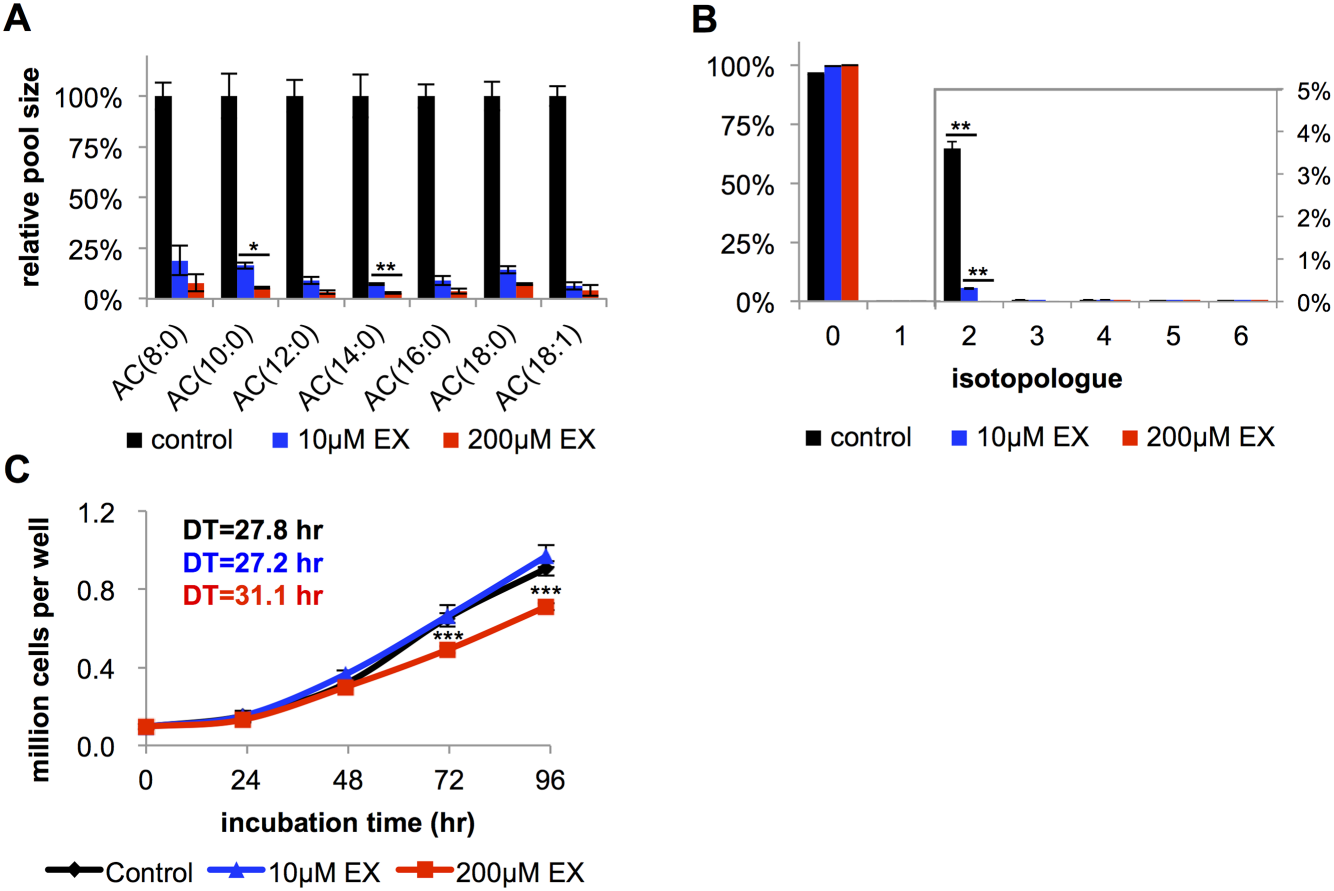
Etomoxir (EX) inhibits most of fatty acid oxidation (FAO) at a 10 μM concentration in BT549 cells but does not affect cellular proliferation until much higher concentrations are used. (A) The pool sizes of acylcarnitines (ACs) decrease by over 80% at 10 μM etomoxir. Additional small decreases are observed at 200 μM etomoxir (*n* = 3). (B) Isotopologue distribution pattern of citrate after BT549 cells were labeled with 100 μM U-^13^C palmitate for 24 hours. The M+2 isotopologue reflects FAO activity (*n* = 3). (C) Growth curve of BT549 cells when treated with vehicle control, 10 μM etomoxir, or 200 μM etomoxir (*n* = 4) (doubling time [DT]). All data are presented as mean ± SEM. *p < 0.05, **p < 0.01, ***p < 0.001.

The first question we sought to address is whether FAO is dispensable in rapidly proliferating cancer cells, such as BT549. We pharmacologically targeted FAO by using the drug etomoxir (ethyl 2-[6-(4-chlorophenoxy)hexyl]oxirane-2-carboxylate), which has been regarded as a specific inhibitor of CPT1 [23, 24]. It is common in cancer studies to use etomoxir at hundreds of micromolar concentrations [5, 15, 18, 25, 26]. Here, we started by considering etomoxir at doses an order of magnitude lower. When BT549 cells were treated with 10 μM etomoxir, we measured over an 80% decrease in acylcarnitine species (the products of CPT1 activity, Fig 1A). Since changes in acylcarnitine levels may not reflect the same change in FAO, we directly assessed FAO by feeding cells uniformly labeled ^13^C-palmitate (U-^13^C palmitate) and measuring the labeling of FAO products. During FAO, U-^13^C palmitate is degraded to ^13^C_2_-acetyl-CoA. This acetyl-CoA then condenses with oxaloacetate in the TCA cycle to produce ^13^C_2_-citrate (the M+2 isotopologue). Upon treatment with 10 μM etomoxir, ^13^C_2_-citrate labeling from U-^13^C palmitate decreased by approximately 90% compared to vehicle controls (Fig 1B). These data demonstrate that 10 μM of etomoxir effectively blocks most of FAO.

Surprisingly, 10 μM etomoxir did not affect the proliferation rate of BT549 cells relative to vehicle controls (Fig 1C). Increasing the concentration of etomoxir by a factor of 10 to 100 μM led to further decreases in acylcarnitine levels and citrate labeling from U-^13^C palmitate, but we still did not observe a statistically significant change in the proliferation rate of BT549 cells (S2A Fig). Comparable results were observed in HeLa cells. When HeLa cells were treated with 100 μM etomoxir, no FAO activity was detected, yet we observed no alteration in proliferation (S2B and S2C Fig). An analysis of 6 additional cell lines produced similar results for B16, 3T3, MCF7, and HS578t cells (S2A Fig). Only 2 cell lines tested (H460 and T47D) showed a statistically significant decrease in proliferation with 100 μM etomoxir treatment. These data suggest that FAO is not an essential source of ATP or NADPH in some cancer cells, such as BT549.

### High concentrations of etomoxir slow cell proliferation

Given that studies evaluating the role of CPT1 in cancer have commonly used concentrations of etomoxir at the hundreds of micromolar or even 1 mM level [15], we next assessed whether higher concentrations of etomoxir affected cell growth. Although 10 μM etomoxir was sufficient to inhibit most of FAO, residual FAO could be further reduced with increasing concentrations of etomoxir. This was reflected by additional small decreases in acylcarnitine pools (Fig 1A) and additional small decreases in the labeling of citrate from U-^13^C palmitate (Fig 1B). Despite the relatively small differences in FAO between 10 and 200 μM etomoxir-treated BT549 cells, we found that 200 μM of etomoxir resulted in a statistically significant reduction in cellular proliferation rate, while 10 μM did not (Fig 1C). These data are consistent with previous reports of the effects of 200 μM etomoxir on BT549 cells [18]. Interestingly, even though no FAO could be measured at 200 μM (Fig 1B), higher concentrations of etomoxir continued to result in further reductions in cell proliferation for BT549 cells (S3A Fig). Similar results were obtained from other cell lines tested (S3B Fig). Taken together, these observations suggest that high concentrations of etomoxir influence proliferation rate independent of FAO.

### Etomoxir causes opposite changes in nutrient utilization at high and low doses

**Fig 2:**
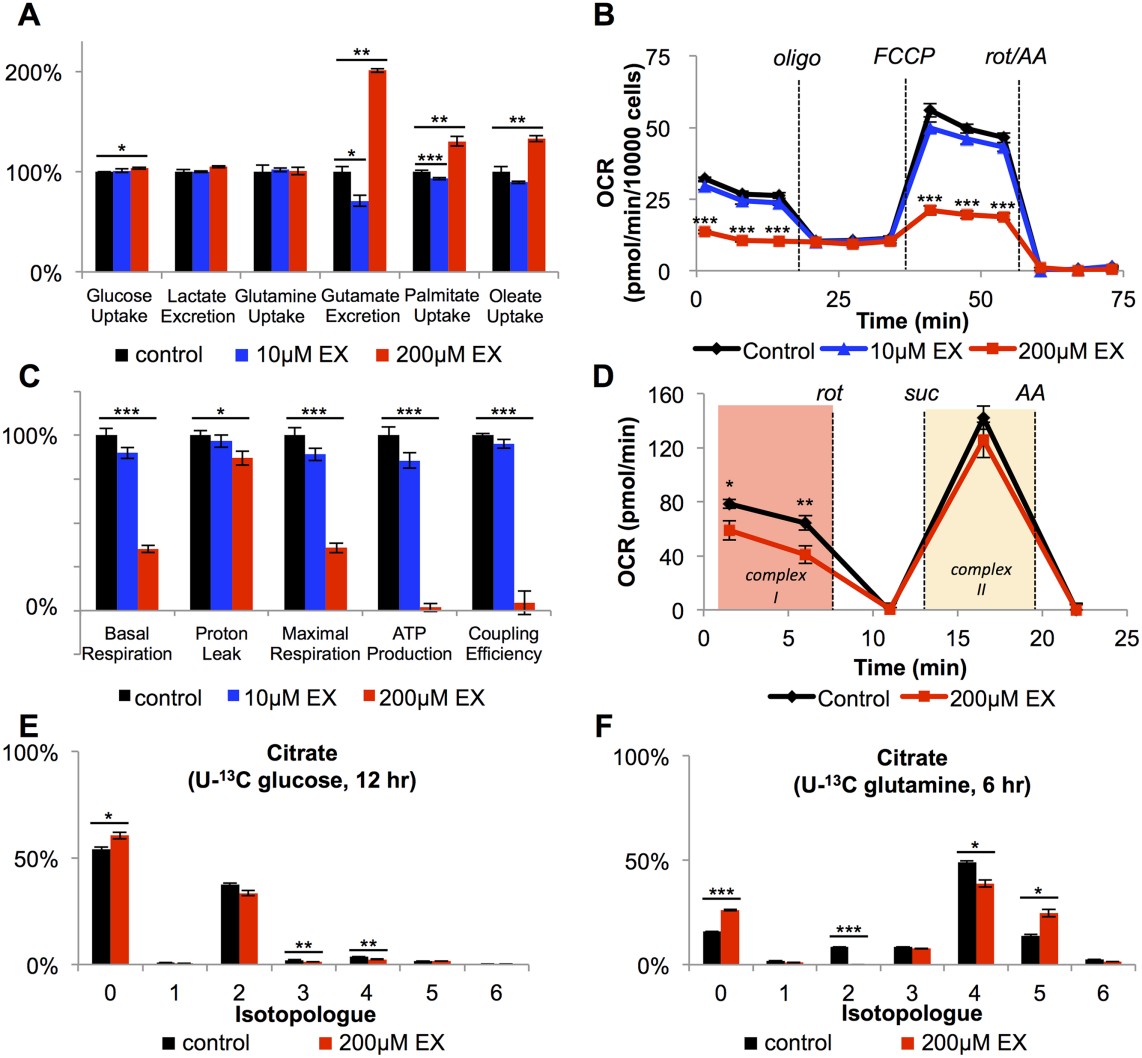
Mitochondrial respiration and nutrient utilization do not show a dose response to etomoxir because 200 μM etomoxir (EX) has an off-target effect on respiratory complex I. (A) Nutrient utilization after BT549 cells were treated with vehicle control, 10 μM etomoxir, or 200 μM etomoxir for 48 hours (*n* = 3). (B) Mitochondrial stress test of whole cells (BT549) after treatment with vehicle control, 10 μM etomoxir, or 200 μM etomoxir for 1 hour (*n* = 4). (C) Measured and calculated parameters of mitochondrial respiration (generated from data in Fig 2B). (D) 200 μM etomoxir leads to changes in state I respiration but does not affect state II respiration, indicating that 200 μM directly inhibits complex I of the electron transport chain (*n* = 3). (E) Isotopologue distribution pattern of citrate after BT549 cells were labeled with U-^13^C glucose for 12 hours (*n* = 3). (F) Isotopologue distribution pattern of citrate after BT549 cells were labeled with U-^13^C glutamine for 6 hours (*n* = 3). All data are presented as mean ± SEM. *p < 0.05, **p < 0.01, ***p < 0.001. The oxygen consumption rate (OCR) was corrected for nonmitochondrial respiration. AA, antimycin A; FCCP, carbonyl cyanide p-trifluoromethoxyphenylhdrazone; oligo, oligomycin; rot, rotenone; suc, succinate.

Since impairing approximately 90% of FAO did not change the rate of BT549 cell proliferation, we hypothesized that these cells might compensate for losses in ATP or NADPH production by increasing the oxidation of metabolic substrates other than fatty acids (e.g., glucose or glutamine). We therefore analyzed cell culture media to evaluate nutrient-uptake and waste-excretion rates of cells treated with etomoxir. Interestingly, when 90% of FAO was inhibited with 10 μM etomoxir, we observed no change in the rate of glucose uptake or lactate excretion (Fig 2A). Instead, with 10 μM etomoxir, we observed a 30% decrease in glutamate excretion. We note that cells treated with 10 μM etomoxir did not alter their glutamine uptake. These data suggest that when FAO is mostly blocked, BT549 cells can possibly compensate for the loss of energy/reducing equivalents by up-regulating glutaminolysis, by which glutamine carbons are fed into the TCA cycle instead of being excreted as glutamate (see S11 Fig, introduced below). Additionally, we observed a 30% decrease in the uptake rate of fatty acids (palmitate and oleate) in etomoxir-treated cells compared to vehicle controls, presumably because drug-treated cells cannot degrade these fatty acids by FAO.

Consistent with our proliferation results, different concentrations of etomoxir resulted in strikingly distinct nutrient utilization profiles that did not correlate with the small differences we observed in FAO. While 10 μM etomoxir did not change glucose uptake or lactate excretion, we observed an increase in glycolysis (indicated by a 3.5% increase in glucose consumption and a 4.8% increase in lactate excretion) when cells were given 200 μM etomoxir (Fig 2A). We also found that in contrast to the decrease in palmitate and oleate uptake we observed in cells treated with 10 μM etomoxir, cells treated with 200 μM etomoxir took up approximately 30% more palmitate and oleate even though these fatty acids could not be oxidized. Most notably, instead of decreasing by 30% as we observed with 10 μM etomoxir treatment, glutamate excretion increased by nearly 2-fold with 200 μM etomoxir (Fig 2A). Considering that glutamine uptake was unaltered, this result suggests that less glutamine carbon is available for oxidation in the TCA cycle at high concentrations of etomoxir (see S11 Fig, introduced below).

### Off-target effects of 200 μM etomoxir on respiratory complex I

Although etomoxir is often assumed to be a specific inhibitor of CPT1, our observations above prompted us to consider other possible off-target activities, particularly at high drug concentrations as are often used in cancer studies [27]. We first examined the OCRs of BT549 cells treated with etomoxir. Cells were assayed in nutrient-rich media containing 25 mM glucose, 4 mM glutamine, 100 μM palmitate, and 100 μM oleate in the presence of vehicle control or etomoxir. Cells were treated with etomoxir for 60 minutes prior to making the oxygen consumption measurements. As we expected based on our nutrient-utilization data and proliferation results, the mitochondrial respiration profiles of cells treated with 10 μM etomoxir were not significantly different from vehicle controls (Fig 2B). With 200 μM etomoxir, however, mitochondrial respiration was significantly impaired. We measured a 65% decrease in basal respiration and a 65% decrease in maximal respiratory capacity after treating cells with 200 μM etomoxir. Moreover, we detected only minimal oxygen-driven ATP production, and the calculated mitochondrial coupling efficiency was therefore determined to be nearly zero (Fig 2C).

Given the impaired mitochondrial respiration that we observed with 200 μM etomoxir treatment in whole cells, we hypothesized that high concentrations of etomoxir might directly inhibit the activity of the electron transport chain. To test this possibility, we isolated intact mitochondria from BT549 cells and measured changes in oxygen consumption upon etomoxir treatment. By using isolated mitochondria instead of whole cells, we could control the availability of substrates for respiration. For this experiment, it is critical to point out that isolated mitochondria were assayed in buffer free of fatty acids, acyl-CoA species, acylcarnitines, and carnitine. Under such conditions, no FAO is occurring, and hence, CPT1 inhibition will not affect respiration. Any change in oxygen consumption upon etomoxir administration can therefore be attributed to off-target effects.

We evaluated mitochondrial respiration in 4 time segments over which various respiratory substrates and inhibitors were added (Fig 2D). The purpose of this experimental design was to distinguish respiration driven by complex I (state I respiration) from respiration driven by complex II (state II respiration). At time zero, mitochondria were provided pyruvate, malate, and ADP. These substrates enable turnover of the TCA cycle and production of NADH. Oxidation of NADH by respiratory complex I drives oxygen consumption. In time segment 2, we added rotenone to the mitochondria. Rotenone inhibits complex I and therefore blocks oxygen consumption under these conditions by preventing the electron transport chain from accepting its only source of electrons. In time segment 3, we provided mitochondria an alternative source of electrons in the substrate succinate. Oxidation of succinate feeds electrons into respiratory complex II of the electron transport chain, which is independent of the rotenone-inhibited complex I and therefore reintroduces oxygen consumption. Finally, in time segment 4, mitochondria were treated with antimycin A. Antimycin A inhibits respiratory complex III, thereby preventing the electron transport chain from oxidizing any of the substrates present. Under these conditions, there is no mitochondrial oxygen consumption. Data from vehicle controls (Fig 2D, black) were as expected.

Next, we independently considered isolated BT549 mitochondria treated with 200 μM etomoxir for 15 minutes. We performed the respiration measurements detailed above over the same 4 time segments. Notably, relative to the vehicle controls, there was a 35% decrease in state I respiration upon etomoxir treatment (Fig 2D, red). However, there was no statistically significant change in state II respiration. In contrast, 10 μM etomoxir resulted in similar OCRs for both state I and state II respiration (S4A Fig). These data suggest that high concentrations (200 μM) of etomoxir inhibit respiratory complex I but do not affect downstream proteins in the electron transport chain. The results also indicate that low concentrations (10 μM) of etomoxir do not have off-target effects on the electron transport chain (S4A Fig). Similar to etomoxir, we note that the complex I inhibitor rotenone also slowed down BT549 cell proliferation when given in culture media (S4B Fig).

We surmised that this off-target effect of 200 μM etomoxir on respiratory complex I might prevent regeneration of NAD^+^ from NADH and hence inhibit the turnover of the TCA cycle, thereby contributing to increased glycolysis and decreased glutaminolysis. Indeed, the intracellular NADH/NAD^+^ ratio was increased in cells treated with 200 μM etomoxir (S5 Fig). To further test our prediction, we fed BT549 cells U-^13^C glucose or U-^13^C glutamine and measured labeling in TCA cycle metabolites. Compared to vehicle controls, labeling of glycolytic intermediates from U-^13^C glucose was slightly increased, while labeling of TCA cycle metabolites from U-^13^C glucose was slightly decreased in cells treated with 200 μM etomoxir (Fig 2E, S6 and S7 Figs). These data are consistent with results shown in Fig 2A, indicating that cells treated with 200 μM etomoxir direct more glucose carbon into lactate instead of aerobic respiration. Although BT549 cells treated with 200 μM etomoxir showed only a modest increase in glycolysis, we note that much larger increases in glycolysis were observed for other cell lines treated with 200 μM etomoxir (S8 Fig). In BT549 cells treated with 200 μM etomoxir, we also observed a decrease in the overall labeling of citrate and other TCA cycle intermediates from U-^13^C glutamine relative to vehicle controls (Fig 2F, S9 Fig). Additionally, the pools of TCA cycle intermediates were decreased, with the exception of α-ketoglutarate, which is the entry point of glutamine into the TCA cycle (S10 Fig). These results are consistent with decreased glutaminolysis, indicated by similar glutamine uptake but increased glutamate excretion (Fig 2A). The relative TCA cycle activity can also be inferred by the ratio of the M+2 isotopologue to the M+4 isotopologue (i.e., M+2/M+4) of malate (S11A Fig). The M+2/M+4 ratio was higher when BT549 cells were treated with 10 μM etomoxir compared to vehicle control, while the M+2/M+4 ratio was lower when BT549 cells were treated with 200 μM etomoxir (S11B Fig). Interestingly, in cells treated with 200 μM etomoxir, we detected increased labeling of the M+5 isotopologue in citrate from U-^13^C glutamine. This result is consistent with a relative increase in the reductive metabolism of glutamine, which is a metabolic signature of cells under hypoxic stress [28].

### CPT1A^KD^ cells have a decreased proliferation rate but increased nutrient uptake

Having established that etomoxir has off-target effects, we chose to use genetic methods to inactivate CPT1. There are 3 subtypes of CPT1 that are encoded by different genes and show tissue-specific distribution [29]. *CPT1B* is expressed in muscle, heart, and adipose tissue and *CPT1C* in neurons, whereas *CPT1A* is more widely expressed and has been previously implicated as a therapeutic target in breast cancer cells [2, 30, 31]. Using siRNA, we knocked down *CPT1A* mRNA levels by >90% relative to scrambled siRNA controls (S12A Fig). All of our assays to phenotype *CPT1A* knockdown (CPT1A^KD^) cells were performed at least 48 hours post transfection and completed within 96 hours, over which time *CPT1A* mRNA levels and protein levels remained greatly reduced (S12B Fig).

**Fig 3:**
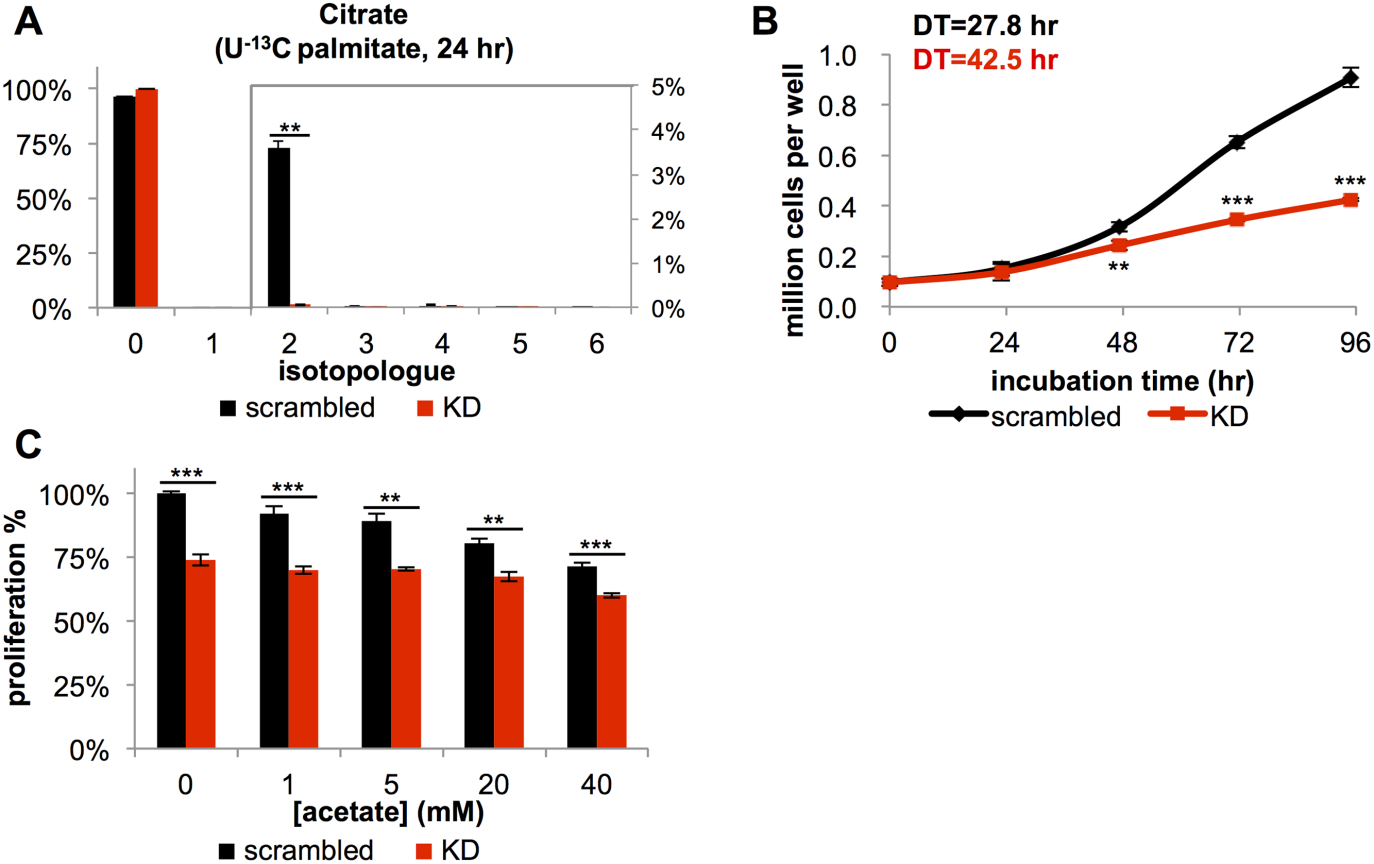
Knockdown of *CPT1A* inactivates most of fatty acid oxidation (FAO) and decreases cellular proliferation. (A) Isotopologue distribution pattern of citrate in BT549 cells with scrambled small interfering RNA (siRNA) (scrambled, black) or after *CPT1A* knockdown (KD, red). Cells were labeled with 100 μM U-^13^C palmitate for 24 hours, starting at 48 hours after siRNA knockdown. The M+2 peak reflects FAO activity (*n* = 3). (B) Growth curve of control and CPT1A^KD^ BT549 cells (*n* = 4) (DT, doubling time). (C) The decrease in cellular proliferation cannot be rescued by various concentrations of acetate (*n* = 5). Data are presented as mean ± SEM. **p < 0.01, ***p < 0.001.

As evidence that knockdown of *CPT1A* blocked transport of fatty acids into the mitochondria, we observed major reductions in the levels of acylcarnitine species (S13 Fig), and we detected no ^13^C-labeled citrate after 24 hours of U-^13^C palmitate labeling (Fig 3A). These data indicated that *CPT1A* knockdown inactivated most of FAO. Notably, CPT1A^KD^ cells had a significantly impaired proliferation rate (Fig 3B), with a 50% increase in doubling time (42.5 hours) compared to control wild-type cells with scrambled siRNA (27.8 hours). Given that the end product of β-oxidation is acetyl-CoA and that acetyl-CoA is readily produced from acetate, acetate supplementation has been shown to rescue cellular functions dependent upon FAO [19]. In our cells, however, impaired proliferation due to *CPT1A* knockdown could not be rescued by acetate supplementation (Fig 3C), suggesting again that CPT1A affects the growth of BT549 cells independent of FAO. Interestingly, supplementation of acetate slightly impaired BT549 cell growth. This could be partially explained by the osmotic effects of sodium, acetate’s counter ion (S14 Fig). We also attempted to rescue the proliferation of knockdown cells by supplementing them with octanoic acid, which can passively diffuse through the inner mitochondrial membrane independent of CPT1 and therefore compensate for impaired FAO [32]. Similar to acetate, supplementing cells with various concentrations of octanoic acid did not restore their proliferation (S15 Fig), further supporting that *CPT1A* knockdown influences cell phenotype independent of FAO.

To rule out the possibility that decreased cell proliferation in *CPT1A* knockdown cells was a result of off-target effects of siRNA, we performed 2 analyses. First, we tested 2 different siRNA sequences and observed comparable protein depletion and growth inhibition in both (S16A and S16B Fig). Given that growth inhibition is a common off-target effect of siRNA, however, we performed a second experiment in which we attempted to rescue CPT1A knockdown cells by overexpressing siRNA-resistant CPT1A protein (CPT1A^resistant^) (S16C Fig). CPT1A^resistant^ protein led to a significant increase in FAO and cellular proliferation rate relative to vector controls (S16D and S16E Fig). Together, these data indicate that decreased proliferation in siRNA-treated cells is due to *CPT1A* loss of function rather than off-target effects.

**Fig 4:**
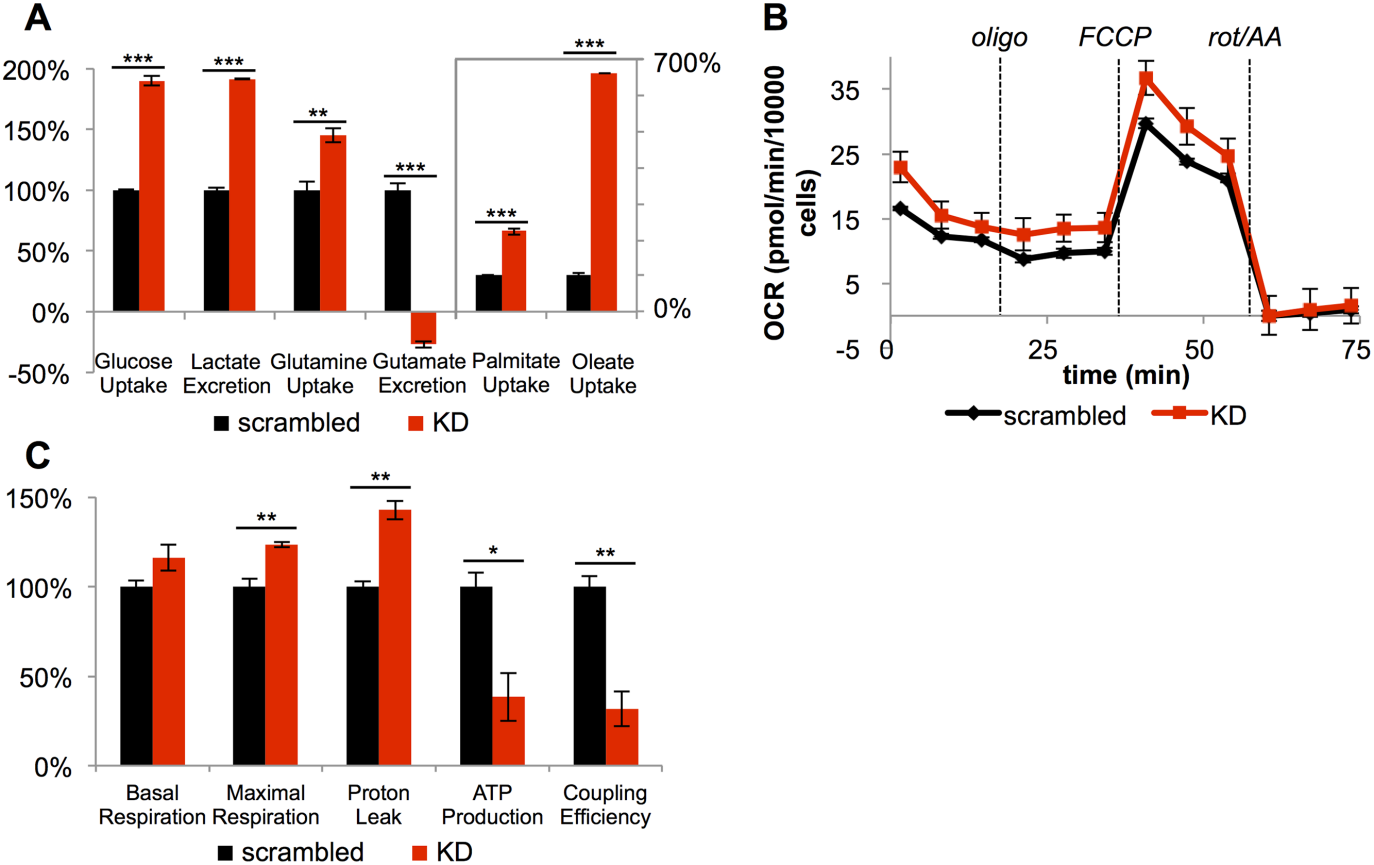
Knockdown of *CPT1A* causes mitochondrial uncoupling. (A) CPT1A^KD^ cells (KD, red) uptake more glucose, glutamine, glutamate, and fatty acids relative to scrambled small interfering RNA (siRNA) controls (scrambled, black). CPT1A^KD^ cells also excrete more lactate (*n* = 4). (B) Mitochondrial stress test for scrambled siRNA controls and CPT1A^KD^ cells (*n* = 3). (C) Measured and calculated mitochondrial respiration parameters (generated from data in Fig 4B). Data are presented as mean ± SEM and normalized to the final number of cells after respiration measurements to account for differences in proliferation. We note that coupling efficiencies are calculated as the ratio of the oxygen consumption rate (OCR) required for ATP production to basal OCR in the same sample and therefore are independent of the sample normalization method. *p < 0.05, **p < 0.01, ***p < 0.001. The OCR was corrected for nonmitochondrial respiration. AA, antimycin A; FCCP, carbonyl cyanide p-trifluoromethoxyphenylhdrazone; oligo, oligomycin; rot, rotenone;.

We also observed changes in nutrient utilization upon *CPT1A* knockdown (Fig 4A). CPT1A^KD^ cells had a nearly 2-fold increase in glucose uptake and lactate production relative to scrambled siRNA controls, indicating a substantial increase in glycolytic flux. Additionally, relative to wild-type cells with scrambled siRNA, CPT1A^KD^ cells had a 2-fold increase in palmitate uptake and a 6.5-fold increase in oleate uptake. Yet, in contrast to cells treated with 200 μM etomoxir, CPT1A^KD^ cells increased their uptake of glutamine by 45% and began uptaking glutamate instead of excreting it (Fig 4A). The increased utilization of glutamine and glutamate carbon suggests increased glutaminolysis and thus increased TCA cycle activity in CPT1A^KD^ cells, whereas data from the etomoxir experiments indicate that 200 μM treated cells have a truncated TCA cycle due to complex I inhibition.

### *CPT1A* knockdown causes mitochondrial uncoupling and changes in mitochondrial morphology

Increases in glycolysis and glutaminolysis are indicative of a change in mitochondrial activity [33]. Thus, we next examined oxygen consumption in whole cells after *CPT1A* knockdown. Unlike cells treated with 200 μM etomoxir, CPT1A^KD^ cells had similar responses to respiratory inhibitors as wild-type cells with scrambled siRNA (Fig 4B). Compared to control cells, however, CPT1A^KD^ cells had a 40% increase in proton leak and a 60% decrease in ATP production. Taken together, CPT1A^KD^ cells had a 70% decrease in mitochondrial coupling efficiency, which compromised their ability to efficiently use respiratory substrates for ATP production. Possibly to compensate for this loss in energy, CPT1A^KD^ cells show increased basal and maximal respiration (Fig 4C). These data are consistent with the observed increase in glucose, glutamine, and glutamate uptake (Fig 4A). We also note that 200 μM etomoxir similarly inhibited respiration in CPT1A^KD^ cells, which is consistent with etomoxir having off-target effects on the respiratory chain independent of CPT1A protein (S17 Fig).

**Fig 5:**
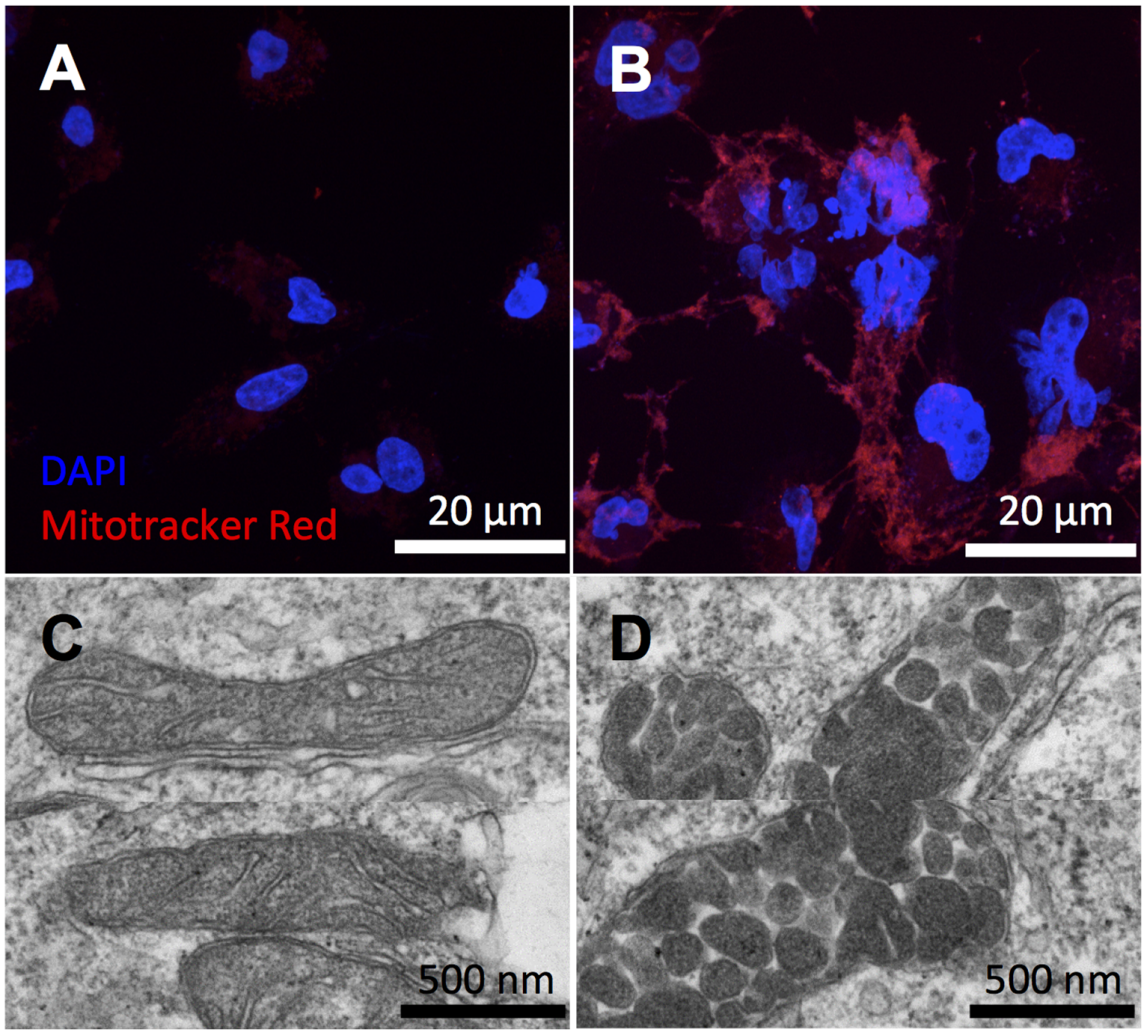
Imaging mitochondrial dysfunction in CPT1A^KD^ cells. (A, B) Mitochondria were stained by Mitotracker red, and nuclei were stained by DAPI. Images from scrambled small interfering RNA (siRNA) controls (A) show less fluorescence intensity of Mitotracker red compared to CPT1A^KD^ cells (B). (C, D) Representative electron microscopy (EM) images of normal mitochondria in wild-type BT549 cells (C) and the abnormal vesicular morphology of mitochondria in CPT1A^KD^ cells (D).

To further examine mitochondrial dysfunction in CPT1A^KD^ cells, we applied fluorescence imaging and electron microscopy (EM). We first stained mitochondria with MitoTracker red, a positively charged fluorescent probe that accumulates as a function of membrane potential. We observed a significant increase in fluorescence intensity from MitoTracker red in CPT1A^KD^ cells relative to controls, suggesting an alteration in mitochondrial membrane potential (Fig 5). Since interpreting this change with respect to increased or decreased mitochondrial membrane potential is complicated by the quenching effects of MitoTracker red at the concentration used, we also compared CPT1A^KD^ and control cells with JC-1 staining [34, 35]. JC-1 accumulates in the mitochondrial matrix as a function of the mitochondrial membrane potential. In the cytosol, JC-1 exists in its monomer form and fluoresces green. Upon its accumulation in the mitochondria, JC-1 forms aggregates that fluoresce red. Accordingly, depolarized mitochondria are characterized by a decrease in the red/green fluorescence intensity ratio [36]. In CPT1A^KD^ cells, we found a decreased ratio of red J-aggregates to green J-monomers relative to control cells (S18 Fig). As expected on the basis of our respiration measurements, these data are consistent with a depolarized mitochondrial membrane due to uncoupling in the CPT1A^KD^ cells. Interestingly, upon *CPT1A* knockdown, we also observed multinucleated cells, which is a signature of cell-cycle arrest [37]. With electron microscopy (EM) imaging, we determined that more than 50% of the mitochondria in CPT1A^KD^ cells had abnormal vesicular morphology compared to the well-defined cristae structure of control cells. Indeed, vesicular cristae shape has been associated with respiratory complex assembly and respiratory efficiency [38–40]. We did not observe abnormal mitochondrial morphology in etomoxir-treated cells (S19 Fig), possibly due to a less complete inactivation of CPT1 compared to knockdowns. We note that although FAO is mostly inhibited in both BT549 cells treated with 200 μM etomoxir (Fig 1B) and in CPT1A^KD^ cells (Fig 3A), the isotopologue distribution patterns of citrate after U-^13^C palmitate labeling cannot be used to compare the level of CPT1A inhibition. This is because 200 μM etomoxir has the off-target effect of inhibiting complex I, which impairs the regeneration of NAD^+^ and thereby influences the oxidative degradation of U-^13^C palmitate.

### Pyruvate and uridine did not restore the proliferation rate of *CPT1A* knockdowns

Pyruvate and uridine enable some cells lacking a functional mitochondrial electron transport chain to proliferate [41, 42]. Thus, we sought to test whether pyruvate and uridine could rescue growth in *CPT1A* knockdowns with dysfunctional mitochondria. When BT549 cells with knocked down *CPT1A* were given pyruvate and uridine, their proliferation rate remained significantly less than that of controls (S20 Fig). These results are consistent with *CPT1A* knockdown cells having a functional electron transport chain that can regenerate oxidized cofactors and suggest that their dysfunctional mitochondria impair cell growth by a different mechanism.

### Evaluating the structural role of CPT1A

Our data suggest that knocking down *CPT1A* affects cell proliferation through a mechanism that is independent of FAO. As one such potential mechanism, we considered the possibility that CPT1A plays an important structural function essential to the integrity of the mitochondrial membrane. To assess this hypothesis, we expressed *CPT1A* having G709E and G710E mutations in BT549 cells. The replacement of glycine residues 709 and 710, which are part of the catalytic site, with glutamate abolishes CPT1A activity (S21A Fig) [43, 44]. We refer to this catalytically dead CPT1A as CPT1A^mutant^. We also note that CPT1A^mutant^ was resistant to any siRNA added to knock down wild-type *CPT1A*. This allowed us to knock down wild-type *CPT1A* in BT549 cells, without affecting *CPT1A*^*mutant*^ expression. We found that expression of CPT1A^mutant^ protein did not rescue cells in which wild-type *CPT1A* had been knocked down. Specifically, expression of *CPT1A*^*mutant*^ did not restore proliferation or mitochondrial membrane potential in wild-type *CPT1A* knockdowns (S21B–S21D Fig). These data do not support a structural role for CPT1A that is independent of FAO.

### Lipidomic analysis reveals alterations in the complex lipids of CPT1A^KD^ cell mitochondria

**Fig 6:**
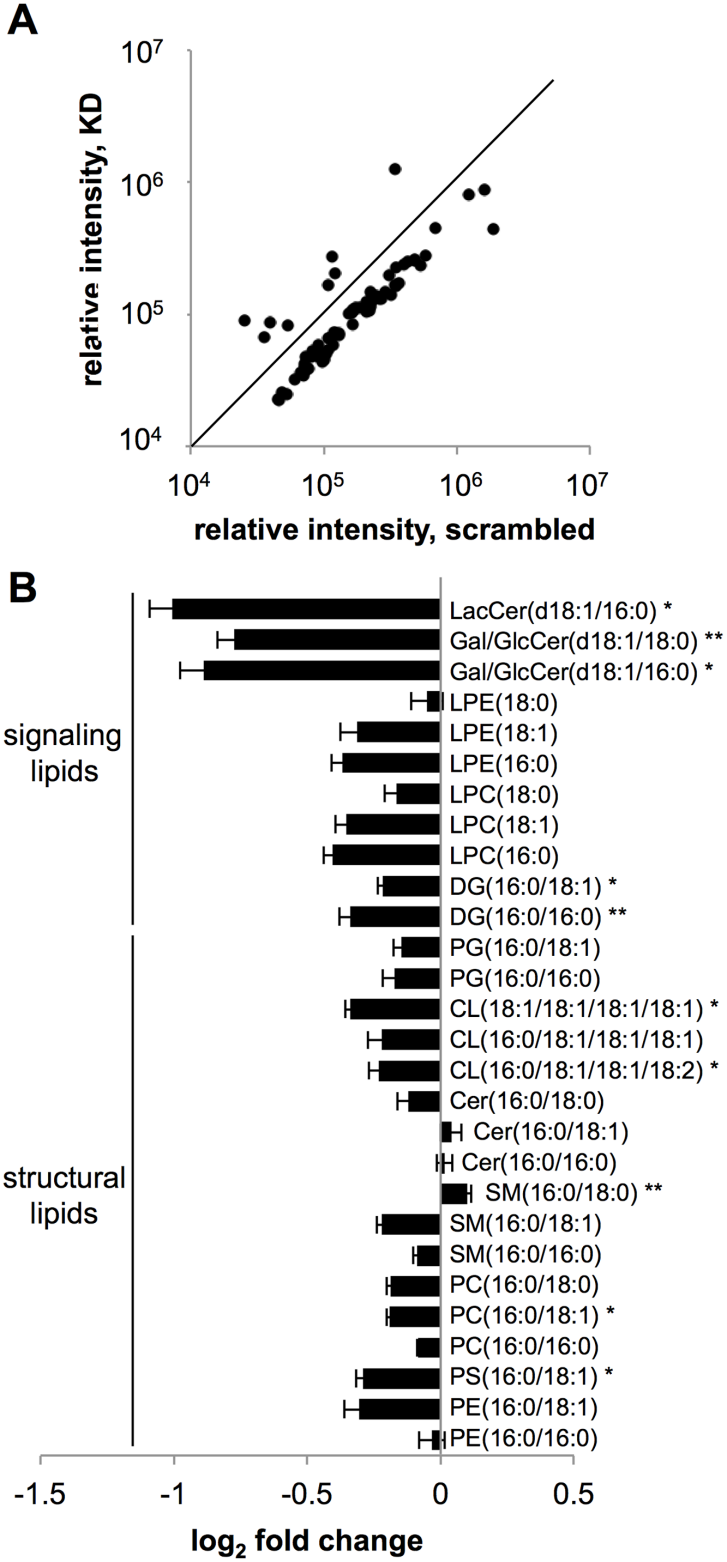
The levels of complex lipids are altered in the mitochondria of CPT1A^KD^ cells. (A) Scatter plot comparing the integrated intensities of 77 lipid species altered between scrambled small interfering RNA (siRNA) controls and CPT1A^KD^ cells. All lipids profiled that showed a fold difference ≥ 1.5, a *p*-value ≤ 0.01, and a signal intensity ≥ 10,000 are displayed. The diagonal line represents the equation *y* = *x*, so that points below the line represent the 66 lipids that decrease in abundance in CPT1A^KD^ cells. (B) The identities and absolute concentrations of dysregulated lipids were determined and the relative differences plotted. Signaling lipids are displayed on top, and structural lipids on bottom. CL, cardiolipin; Cer, ceramide; DG, diacylglycerol; Gal/GlcCer, galactosyl/glucosylceramide; KD, knockdown; LacCer, lactosylceramide; LPC, lysophosphatidylcholine; LPE, lysophosphatidylethanolamine; PC, phosphatidylcholine; PE, phosphatidylethanolamine; PG, phosphatidylglycerol; PS, phosphatidylserine; SM, sphingomyelin. Data are presented as mean ± SEM (*n* = 3). *p < 0.05, **p < 0.01.

As another mechanism for how CPT1A may influence cell proliferation independent of FAO, we considered the possibility that CPT1A mediates transport of long-chain fatty acids into the mitochondria for anabolic purposes. That is, instead of oxidizing long-chain fatty acids transported into the mitochondria by CPT1A for energy, we hypothesized that the carnitine shuttle provides an indispensable source of fatty acids to synthesize complex lipids during cellular proliferation [45]. To test our hypothesis, we isolated mitochondria from CPT1A^KD^ and wild-type cells and applied lipidomic profiling to quantitate differences in mitochondrial lipids. Consistent with our prediction, many complex lipid species had decreased levels in CPT1A^KD^ cells relative to wild-type cells (Fig 6A). In our untargeted profiling experiment, 87% of the dysregulated lipids were decreased (see S1 Table). We then quantified the change in concentrations of these altered lipid features, which included complex structural lipids such as phospholipids, sphingolipids, and cardiolipins (Fig 6B). We also observed a nearly 2-fold decrease in complex signaling lipids such as lactosylceramide and glucosyl/galactosylceramides. Smaller decreases were found in other signaling lipids such as lysophospholipids and diacylglycerols. Whether they are a direct consequence of limited long-chain fatty acid availability or a downstream consequence of altered mitochondrial metabolism, these data suggest that CPT1A plays a role in regulating the levels of mitochondrial lipids.

## Discussion

In recent years, multiple cancers have been found to have increased expression of *CPT1* and/or sensitivity to CPT1 inhibition [6, 9]. In the conventional textbook picture of mammalian metabolism, CPT1 commits long-chain fatty acids to catabolic oxidation [46]. Thus, increased expression of *CPT1* and/or sensitivity to CPT1 inhibition has been assumed to represent a demand for FAO and the ATP or NADPH provided. Our work here reveals 2 complications with this interpretation: (1) pharmacological inhibition of CPT1 with high concentrations of etomoxir, as is often used in cancer studies, leads to off-target effects, and (2) CPT1 influences the proliferation of several cancer cell lines independent of FAO.

Treatment of BT549 breast cancer cells as well as several other cancer cell lines with 200 μM etomoxir significantly slowed cell proliferation, which is consistent with previous studies [18]. However, decreased cell proliferation at 200 μM etomoxir is not a result of inhibiting the primary target of etomoxir (i.e., CPT1). Rather, 200 μM etomoxir inhibits complex I of the electron transport chain (an off-target effect) and leads to decreased cell proliferation independent of FAO. We note that 10 μM etomoxir efficiently blocked 90% of FAO and did not exhibit off-target effects on respiration; however, 10 μM etomoxir did not reduce BT549 cell proliferation. When most of FAO was inhibited with 10 μM etomoxir, BT549 cells adjust their uptake and utilization of other nutrients to compensate for the loss of FAO. These data indicate that FAO provides a dispensable source of ATP and reducing equivalents under standard cell-culture conditions.

FAO generates acetyl-CoA, FADH_2_, NADH, ATP, and potentially cytosolic NADPH. Importantly, all of these products can be derived from other nutrient sources without using CPT1. Glucose, for example, can provide cytosolic NADPH via the pentose phosphate pathway and acetyl-CoA from glycolysis and the pyruvate dehydrogenase complex. FADH_2_, NADH, and ATP can be obtained from the oxidation of glucose carbon through the TCA cycle. Similarly, reducing equivalents and ATP can be readily derived from glutamine [47]. Thus, while the products of FAO are highly valuable to a cell and may serve as a major energy source, they are not unique to the FAO pathway. Our results suggest that some cells, such as BT549, can therefore compensate for the loss of FAO by adjusting nutrient uptake and utilization.

Inhibiting approximately 90% of FAO by pharmacological inhibition of CPT1 did not affect the proliferation rate of BT549 cells, but genetic knockdown of *CPT1A* did. Moreover, genetic knockdown of *CPT1A* altered mitochondrial morphology and caused mitochondrial uncoupling, while pharmacological inhibition of CPT1 did not. These data together with the observations that acetate and octanoic acid did not rescue *CPT1A* knockdowns indicate that CPT1A has a function affecting cell proliferation that is independent of its role in FAO. We first considered a structural function of CPT1A as a scaffolding protein. However, expression of a catalytically dead CPT1A in BT549 cells in which wild-type *CPT1A* had been knocked down did not restore mitochondrial membrane potential.

**Fig 7:**
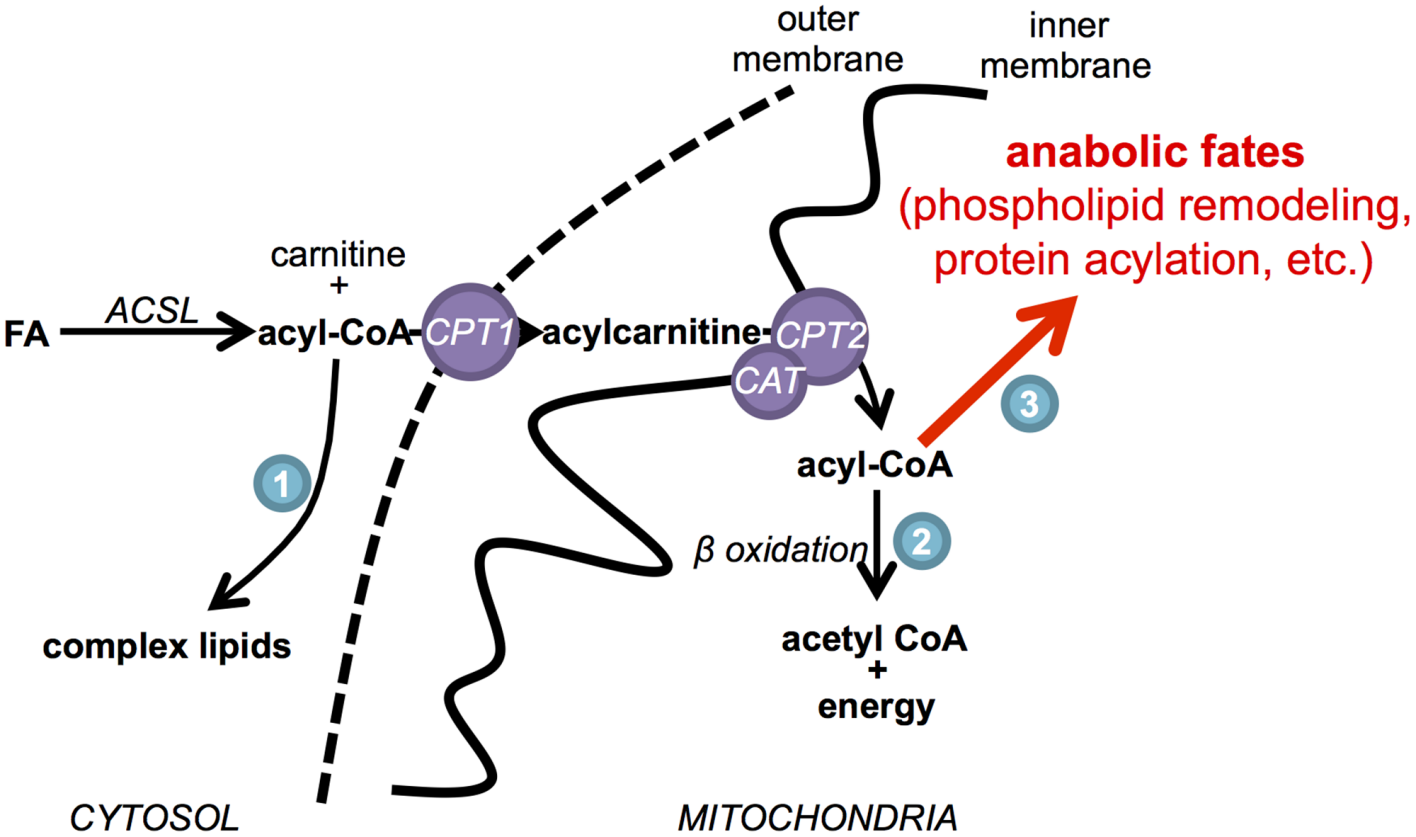
Model for the anabolic role of carnitine palmitoyltransferase I (CPT1) in mitochondrial metabolism. Acyl-CoA species have anabolic fates in the cytosol (1), in addition to catabolic (2) and anabolic (3) fates in the mitochondrial matrix (e.g., phospholipid sidechain remodeling and protein acylation). ACSL, acyl-CoA synthetase; CAT, carnitine-acylcarnitine translocase; CPT1, carnitine palmitoyltransferase I; CPT2, carnitine palmitoyltransferase II; FA, fatty acid.

As another possible function of CPT1 that is independent of FAO, we considered the need to use CPT1 for purposes other than catabolic oxidation of lipids. Without CPT1, cells cannot transport long-chain fatty acids into mitochondria, and therefore, downstream mitochondrial pathways using these substrates are impaired (Fig 7). Sources of long-chain fatty acids (or long-chain fatty acyl-CoAs) inside the mitochondria that do not rely on the CPT1 transport system are limited [48–50]. Complex lipids synthesized in the endoplasmic reticulum can be transported to the mitochondria and deacylated to make long-chain fatty acids [51, 52], or long-chain fatty acids can be generated in the mitochondrial matrix by type II mitochondrial fatty acid synthesis, a pathway that resembles fatty acid synthesis in bacteria [53]. Although the fates of long-chain fatty acids generated by these processes remain poorly understood, disrupting mitochondrial fatty acid synthesis slows cell growth, influences mitochondrial phospholipid composition, and alters mitochondrial morphology [54–58], phenotypes which are highly consistent with those that we observed here with *CPT1A* knockdown. One possible explanation for these findings is that long-chain fatty acids generated in mitochondria are involved in phospholipid side-chain remodeling [54]. The de novo synthesis of cardiolipin in the mitochondria, for example, is followed by cycles of deacylation and reacylation. This remodeling process is essential to mitochondrial structure and function and, at least in part, uses acyl-CoA substrates in the mitochondrial matrix [59, 60]. Another possible demand for long-chain fatty acids in the mitochondria is protein acylation, which may be used for protein anchoring, cell signaling, or protein trafficking. Although acylation of mitochondrial proteins remains largely unexplored, many mitochondrial proteins have been shown to be modified with long acyl chains in the mitochondrial matrix [61, 62]. It is important to note that any anabolic demand for long-chain fatty acids transported by CPT1A in BT549 cells is likely to be low, since pharmacologically inhibiting most of CPT1 activity with low concentrations of etomoxir does not result in decreased cell proliferation or mitochondrial dysfunction. Interestingly, the demand for mitochondrial fatty acid synthesis is also low, but its disruption similarly results in decreased cell proliferation and mitochondrial dysfunction [54]. Our results therefore suggest that, like mitochondrial fatty acid synthesis, the CPT1 system may provide an indispensable source of long-chain fatty acids in the mitochondria to support processes that do not demand much carbon (such as phospholipid remodeling and protein acylation) but are essential to healthy mitochondrial function and cancer cell proliferation. We also point out that the results obtained for the cancer cells studied here are unlikely to be generalizable to all cancer cells; however, they demonstrate that additional evidence independent of CPT1 is necessary to implicate FAO as an antitumor target.

## Supporting information

S1 Data Numerical data and statistical analysis for results shown in Figs 1A, 1B and 1C, 2A, 2B, 2C, 2D, 2E and 2F, 3A, 3B and 3C, 4A, 4B and 4C, 6A and 6B. (XLSX)

S1 Fig Data from manual cell counting and an MTT assay show comparable decreases in cell proliferation after BT549 cells were treated with 200 μM etomoxir (EX) for 48 hours (*n* = 4). Data are presented as mean ± SEM. **p < 0.01.
(TIFF)

S2 Fig Effects of etomoxir (EX) treatment on various cell lines. (A) Relative proliferation rates of different cell lines treated with 100 μM etomoxir for 48 hours compared to cells treated with vehicle control (*n* = 5). (B) Acylcarnitine levels decrease in HeLa cells after etomoxir treatment (*n* = 3). (C) Isotopologue distribution pattern of citrate after HeLa cells were labeled with 100 μM U-^13^C palmitate for 24 hours. The M+2 isotopologue peak reflects fatty acid oxidation (FAO) activity (*n* = 3). Data are presented as mean ± SEM. *p < 0.05.
(TIFF)

S3 Fig Proliferation rates of BT549 and other cancer cells at high concentrations of etomoxir (EX). (A) The proliferation rate of BT549 cells decreases as etomoxir concentrations increase (*n* = 5). Cells were treated with etomoxir for 48 hours. (B) Other cancer cell lines tested show decreased proliferation after 200 μM etomoxir treatment for 48 hours (*n* = 5). Data are presented as mean ± SEM. *p < 0.05, **p < 0.01, ***p < 0.001.
(TIFF)

S4 Fig Off-target effect of 200 μM etomoxir (EX) on the electron transport chain. (A) Two hundred μM etomoxir inhibits state I respiration (corresponding to complex I), while 10 μM etomoxir does not (*n* = 3). The 37% difference between basal respiration and 200 μM etomoxir treatment is smaller than the 65% difference observed in Fig 2B, likely due to the absence of fatty acid oxidation and the reduced basal respiration of isolated mitochondria [63, 64]. (B) The complex I inhibitor, rotenone, slows down BT549 cell proliferation at various concentrations (*n* = 5). Data are presented as mean ± SEM. *n*.*s*., not statistically significant,**p < 0.01, ***p < 0.001.
(TIFF)

S5 Fig Intracellular NADH/NAD^+^ ratios in vehicle control cells and cells treated with 200 μM etomoxir for 48 hours (*n* = 3). Data are presented as mean ± SEM. *p < 0.05.
(TIFF)

S6 Fig Isotopologue distribution patterns of glycolytic intermediates from U-^13^C glucose after 200 μM etomoxir (EX) treatment. BT549 cells were treated with vehicle control or 200 μM etomoxir for 48 hours and then labeled with U-^13^C glucose for 12 hours in the presence of vehicle control or etomoxir (*n* = 3). Data are presented as mean ± SEM.
(TIFF)

S7 Fig Decreased labeling of tricarboxylic acid (TCA) cycle intermediates from U-^13^C glucose after 200 μM etomoxir (EX) treatment. BT549 cells were treated with vehicle control or 200 μM etomoxir for 48 hours and then labeled with U-^13^C glucose for 12 hours in the presence of vehicle control or etomoxir (*n* = 3). Data are presented as mean ± SEM.
(TIFF)

S8 Fig Etomoxir at 200 μM increases glucose uptake and lactate excretion in HeLa and MCF7 cells. Data are presented as mean ± SEM. **p < 0.01, ***p < 0.001.
(TIFF)

S9 Fig Decreased labeling of tricarboxylic acid (TCA) cycle intermediates from U-^13^C glutamine after 200 μM etomoxir (EX) treatment. BT549 cells were treated with vehicle control or 200 μM etomoxir for 48 hours and then labeled with U-^13^C glutamine for 6 hours in the presence of vehicle control or etomoxir (*n* = 3). Data are presented as mean ± SEM.
(TIFF)

S10 Fig The relative pool sizes of citrate, malate, and aspartate decreased, while the relative pool size of α-ketoglutarate (αKG) increased after cells were treated with 200 μM etomoxir (EX) for 48 hours (*n* = 3). Pool sizes were normalized to cell dry mass, and deuterated phenylalanine (D8) was used as an internal standard. Data are presented as mean ± SEM. *p < 0.05, ***p < 0.001.
(TIFF)

S11 Fig The M+2/M+4 isotopologue ratio of malate indicates an increase in tricarboxylic acid (TCA) cycle activity with 10 μM etomoxir (EX) treatment and a decrease in TCA cycle activity with 200 μM etomoxir treatment. (A) Schematic showing the origin of the M+2 and M+4 isotopologues in the TCA cycle from U-^13^C glutamine. Red circles represent ^13^C-labeled carbon, and grey circles represent unlabeled carbon. (B) Isotopologue distribution pattern of malate after labeling with U-^13^C glutamine for 6 hours (*n* = 3). Data are presented as mean ± SEM. **p < 0.01, ***p < 0.001.
(TIFF)

S12 Fig *CPT1A* expression level and CPT1A protein level after small interfering RNA (siRNA) knockdown. (A) *CPT1A* mRNA levels were determined by quantitative reverse transcription PCR (qRT-PCR) (normalized to an HPRT endogenous control) (*n* = 3). (B) Western blot analysis of cell lysate after siRNA knockdown for 48, 72, or 96 hours. β-tubulin was used as a loading control. Scrambled siRNA was used as negative control (control).
(TIFF)

S13 Fig Acylcarnitine levels decreased by over 80% in CPT1A^KD^ cells. The acylcarnitine levels of long-chain fatty acids decreased by over 90%. Data are from cells harvested at 72 hours post small interfering RNA (siRNA) transfection (*n* = 3). Data are presented as mean ± SEM. *p < 0.05, **p < 0.01.
(TIFF)

S14 Fig High concentrations of sodium chloride (NaCl) slightly impaired BT549 cell proliferation (*n* = 5). Data are presented as mean ± SEM. **p < 0.01, ***p < 0.001.
(TIFF)

S15 Fig The decrease in BT549 cell proliferation after *CPT1A* knockdown cannot be rescued by various concentrations of octanoic acid (*n* = 5). Data are presented as mean ± SEM. **p < 0.01, ***p < 0.001.
(TIFF)

S16 Fig Decreased proliferation of BT549 cells is caused by *CPT1A* knockdown. (A) Two different dicer-substrate short interfering RNA (DsiRNA) sequences (see S1 Text) were evaluated individually or as a pool (*n* = 5). They both resulted in a comparable decrease in BT549 cell proliferation. (B) Western blot analysis of cell lysates after small interfering RNA (siRNA) knockdown for 72 hours shows that both siRNA sequences resulted in decreased expression of CPT1A protein. (C) Western blot analysis of lysates from whole cells and isolated mitochondria shows that only some overexpressed CPT1A localized to mitochondria. (D) Overexpression of siRNA-resistant *CPT1A* (CPT1A^resistant^) protein partially rescues the proliferation of CPT1A^KD^ cells (*n* = 5). The DNA sequence for CPT1A^resistant^ is shown in S1 Text. The control vector was the same vector construct, but it expressed green fluorescent protein (GFP) instead of CPT1A. (E) Isotopologue distribution pattern of citrate after BT549 cells were labeled with 100 μM U-^13^C palmitate for 24 hours following a 72-hour knockdown and 48-hour overexpression. The M+2 isotopologue reflects fatty acid oxidation (FAO) activity. In CPT1A^KD^ cells that overexpressed siRNA-resistant *CPT1A*, FAO activity was restored. All data are presented as mean ± SEM. **p < 0.01, ***p < 0.001.
(TIFF)

S17 Fig Mitochondrial stress test of CPT1A^KD^ whole cells (BT549) after treatment with vehicle control or 200 μM etomoxir for 1 hour (*n* = 3). All data are presented as mean ± SEM. **p < 0.01, ***p < 0.001. The oxygen consumption rate (OCR) was corrected for nonmitochondrial respiration.
(TIFF)

S18 Fig JC-1 staining indicates that CPT1A^KD^ cells have depolarized mitochondria. (A) After cells were treated with scrambled small interfering RNA (siRNA) or *CPT1A* siRNA for 72 hours, mitochondria were stained with JC-1. Red fluorescence of J-aggregates was detected by excitation with the 514-nm argon-ion laser source, and green fluorescence of J-monomers was detected with the 543-nm helium neon laser source. (B) The absolute fluorescence intensities of several representative images were quantified. The relative ratio of red J-aggregates to green J-monomers in scrambled siRNA controls and CPT1A^KD^ cells was plotted (*n* = 5). Data are presented as mean ± SEM. *p < 0.05.
(TIFF)

S19 Fig BT549 cells exhibit normal mitochondrial morphology after treatment with etomoxir for 48 hours. Etomoxir concentrations of (A) 10 μM and (B) 200 μM were tested. Mitochondria were stained by Mitotracker red, and nuclei were stained by DAPI. (C, D) Representative electron microscopy (EM) images of mitochondria from cells treated with (C) 10 μM etomoxir or (D) 200 μM etomoxir.
(TIFF)

S20 Fig Proliferation of CPT1A^KD^ cells could not be rescued by supplementation of various concentrations of uridine and pyruvate (*n* = 5). Data are presented as mean ± SEM. *p < 0.05, **p < 0.01, ***p < 0.001.
(TIFF)

S21 Fig Overexpression of a catalytically dead CPT1A protein (CPT1A^mutant^) did not rescue the proliferation or restore mitochondrial membrane potential of CPT1A^KD^ cells. (A) The isotopologue distribution pattern of citrate after BT549 cells were labeled with 100 μM U-^13^C palmitate for 24 hours following a 72-hour knockdown and 48-hour overexpression. The M+2 isotopologue reflects fatty acid oxidation (FAO). As expected, in CPT1A^KD^ cells that overexpressed a catalytically dead CPT1A, FAO was not restored. We note that the catalytically dead CPT1A protein is also resistant to knockdown by the small interfering RNA (siRNA) used. The control vector was the same as the vector construct, but it expressed green fluorescent protein (GFP) instead of CPT1A. (B) Overexpression of a catalytically dead CPT1A protein did not restore the proliferation of CPT1A^KD^ cells (*n* = 5). (C) Mitochondria were stained by Mitotracker red, and nuclei were stained by Hoechst 33342. Quantitation of fluorescence intensity is shown in panel (D). (D) Total fluorescence intensity of Mitotracker red from 3 representative fields taken at 20× (*n* = 3). All data are presented as mean ± SEM. *n*.*s*., not statistically significant, *p < 0.05.
(TIFF)

S1 Table Dysregulated features identified by untargeted profiling. (DOCX)

S1 Text Sequences for dicer-substrate short interfering RNA (DsiRNA), CPT1A^resistant^, and CPT1A^mutant^. (DOCX)

## Abbreviations

ADP: adenosine diphosphate
CPT1: carnitine palmitoyltransferase I
DsiRNA: dicer-substrate short interfering RNA
FAO: fatty acid oxidation
OCR: oxygen consumption rate
siRNA: small interfering RNA
TCA: tricarboxylic acid
