# Supplementary figures and images for: Identifying off-target effects of etomoxir reveals that carnitine palmitoyltransferase I is essential for cancer cell proliferation independent of β-oxidation
Source: PLoS Biol. 2018 Mar 29;16(3):e2003782. doi: 10.1371/journal.pbio.2003782 (PMC5892939; doi:10.1371/journal.pbio.2003782)

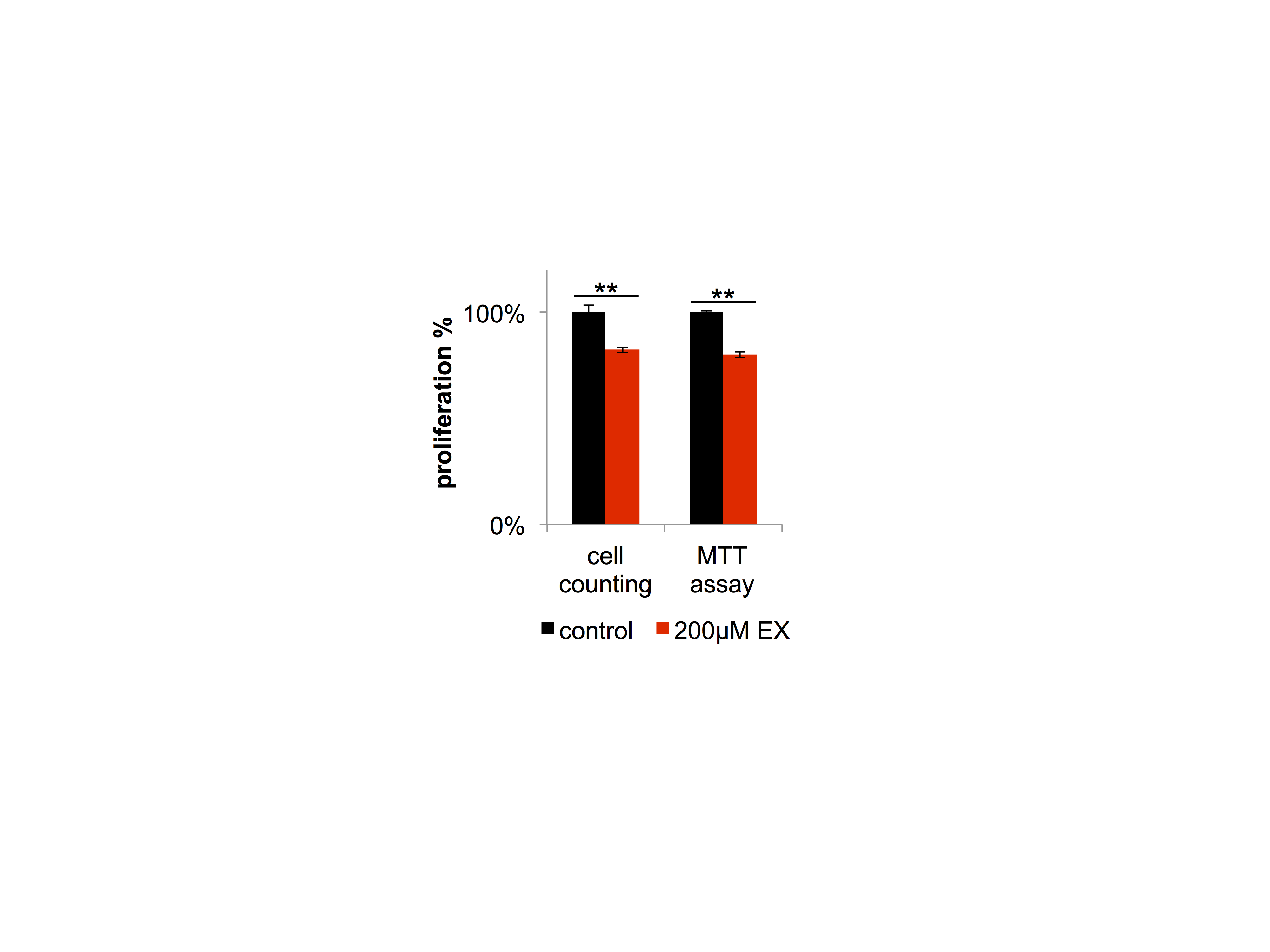

Supplement: S1 Fig — Data are presented as mean ± SEM. **p < 0.01. (TIFF) [file pbio.2003782.s002.tiff]

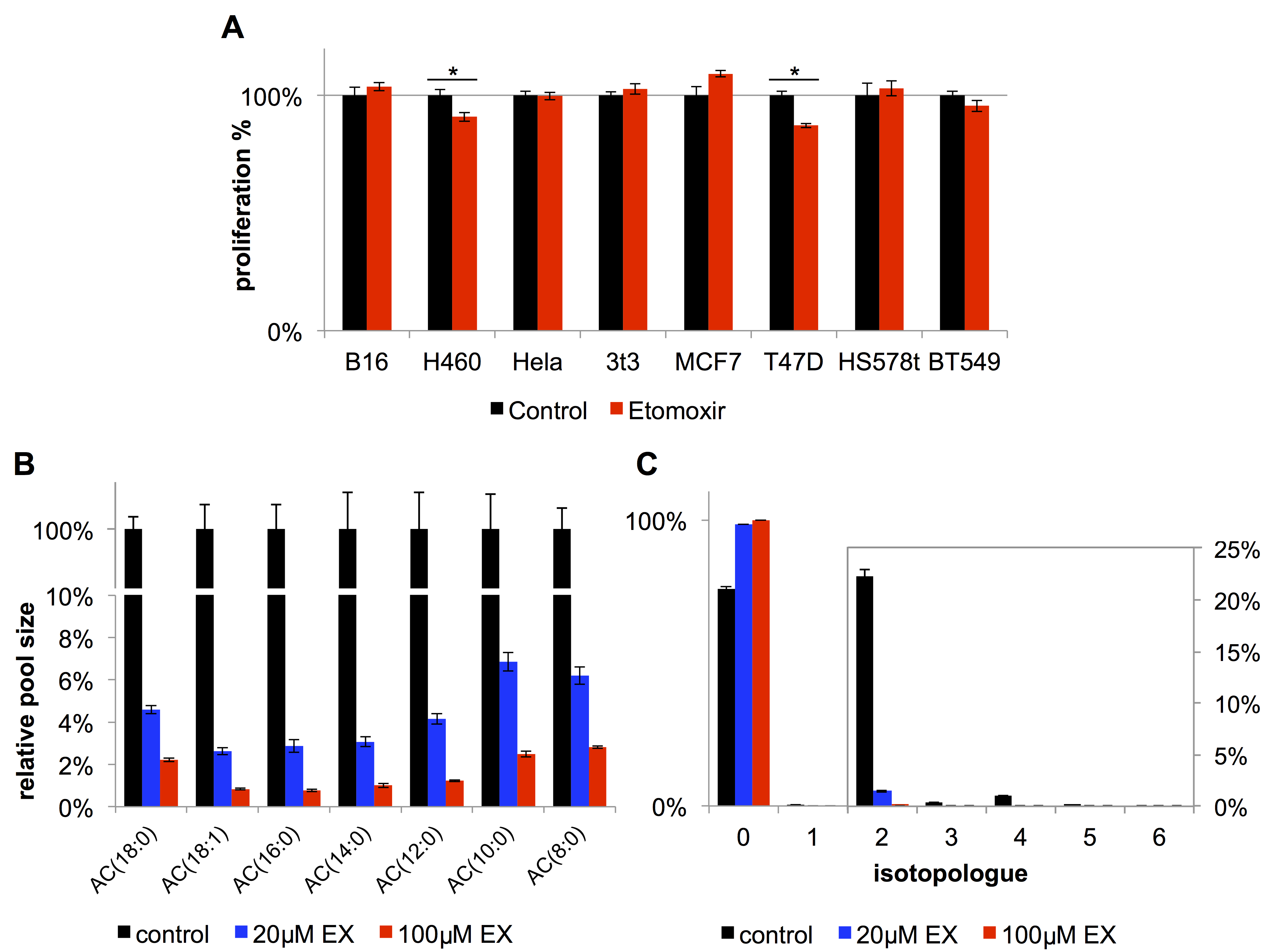

Supplement: S2 Fig — (A) Relative proliferation rates of different cell lines treated with 100 μM etomoxir for 48 hours compared to cells treated with vehicle control (n = 5). (B) Acylcarnitine levels decrease in HeLa cells after etomoxir treatment (n = 3). (C) Isotopologue distribution pattern of citrate after HeLa cells were labeled with 100 μM U-13C palmitate for 24 hours. The M+2 isotopologue peak reflects fatty acid oxidation (FAO) activity (n = 3). Data are presented as mean ± SEM. *p < 0.05. (TIFF) [file pbio.2003782.s003.tiff]

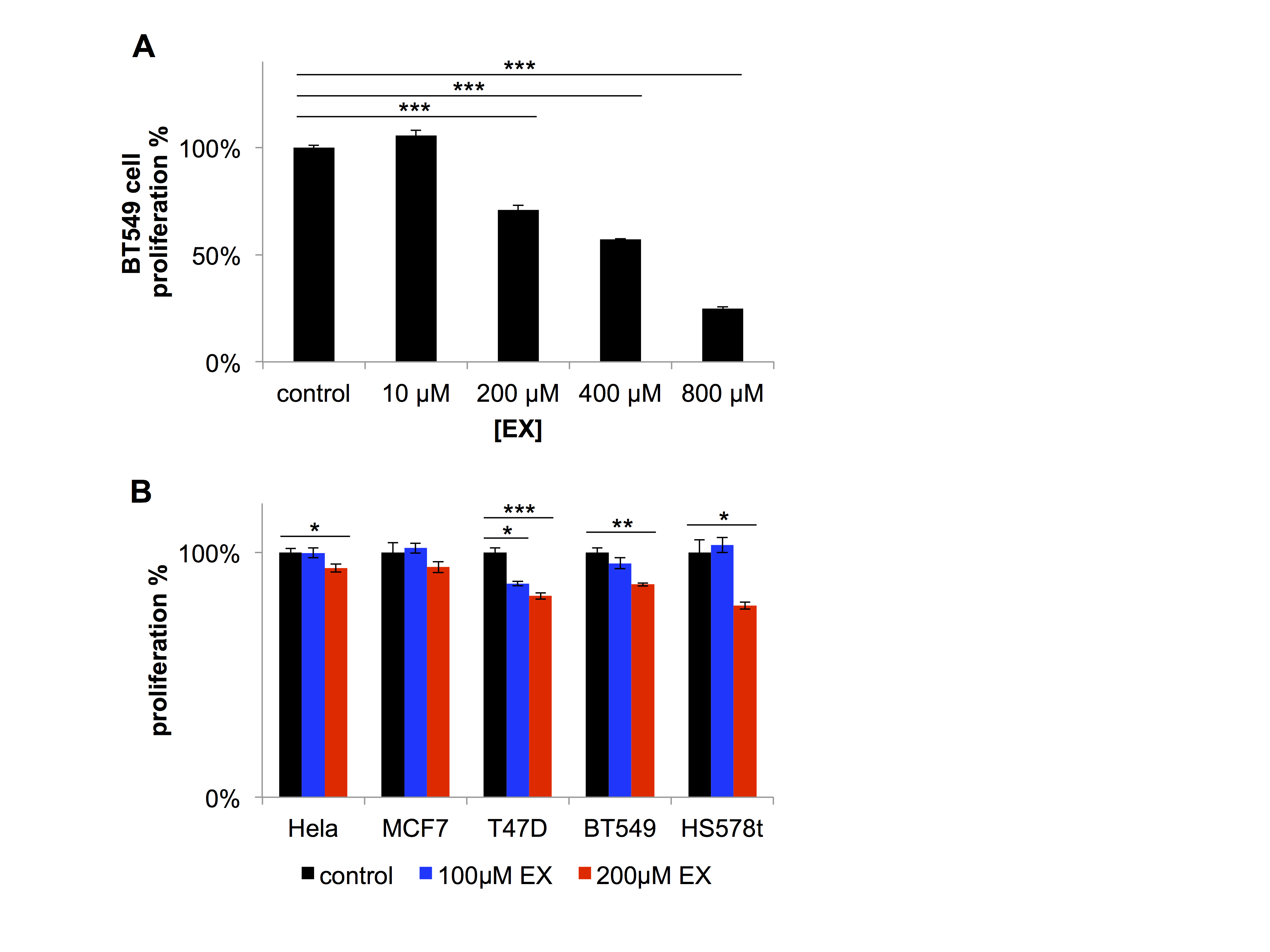

Supplement: S3 Fig — (A) The proliferation rate of BT549 cells decreases as etomoxir concentrations increase (n = 5). Cells were treated with etomoxir for 48 hours. (B) Other cancer cell lines tested show decreased proliferation after 200 μM etomoxir treatment for 48 hours (n = 5). Data are presented as mean ± SEM. *p < 0.05, **p < 0.01, ***p < 0.001. (TIFF) [file pbio.2003782.s004.tiff]

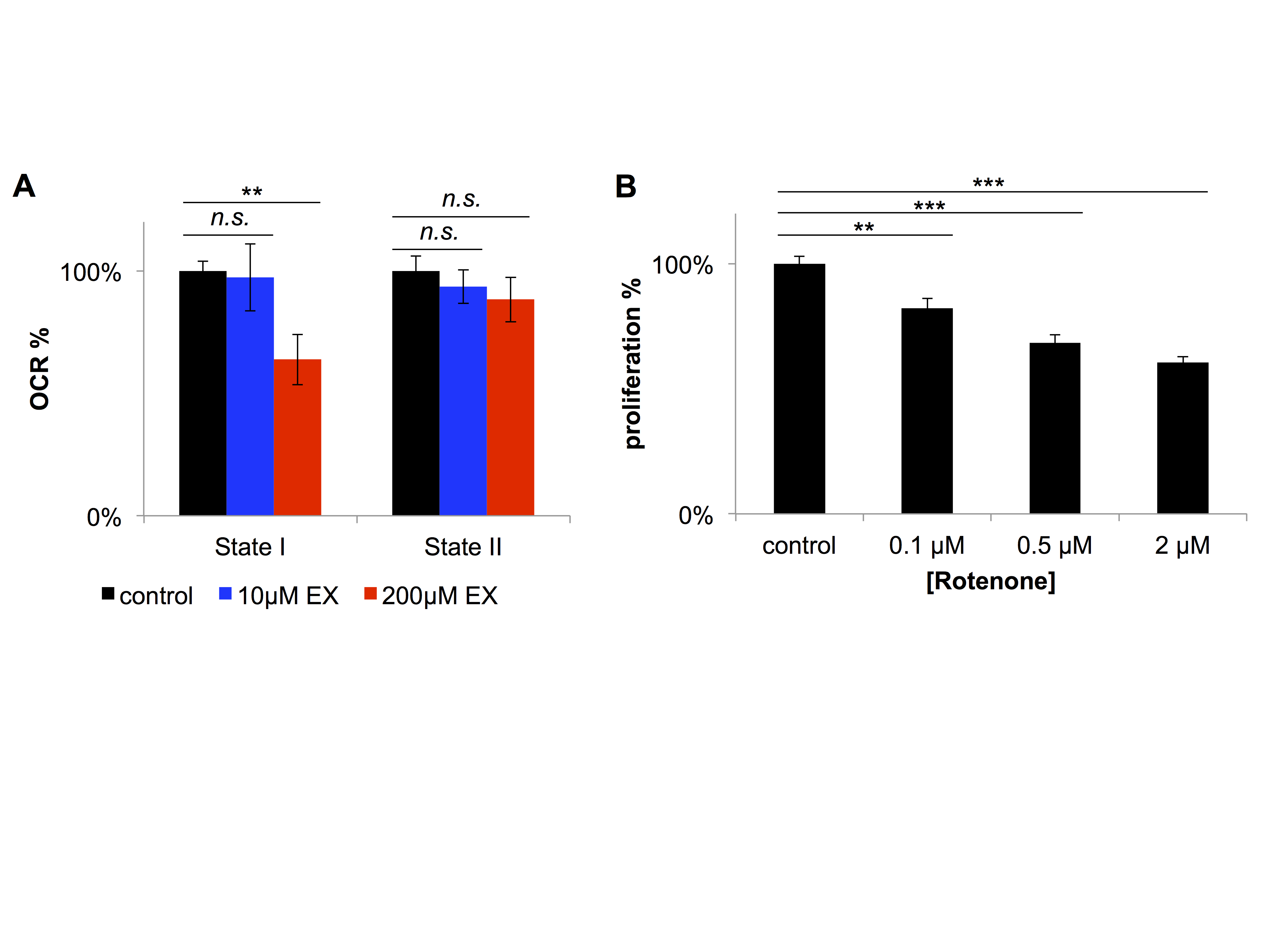

Supplement: S4 Fig — (A) Two hundred μM etomoxir inhibits state I respiration (corresponding to complex I), while 10 μM etomoxir does not (n = 3). The 37% difference between basal respiration and 200 μM etomoxir treatment is smaller than the 65% difference observed in Fig 2B, likely due to the absence of fatty acid oxidation and the reduced basal respiration of isolated mitochondria [63, 64]. (B) The complex I inhibitor, rotenone, slows down BT549 cell proliferation at various concentrations (n = 5). Data are presented as mean ± SEM. n.s., not statistically significant,**p < 0.01, ***p < 0.001. (TIFF) [file pbio.2003782.s005.tiff]

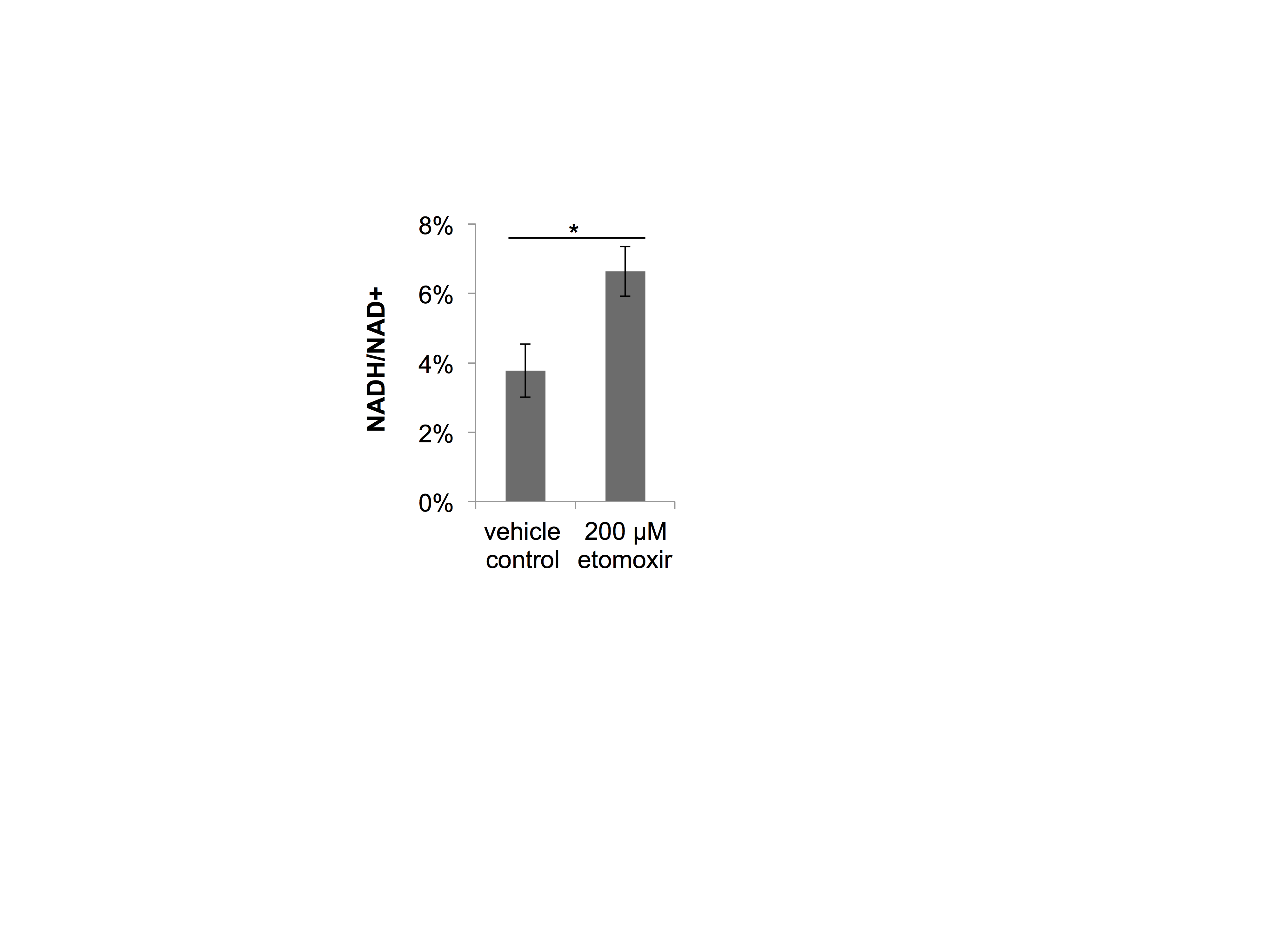

Supplement: S5 Fig — Data are presented as mean ± SEM. *p < 0.05. (TIFF) [file pbio.2003782.s006.tiff]

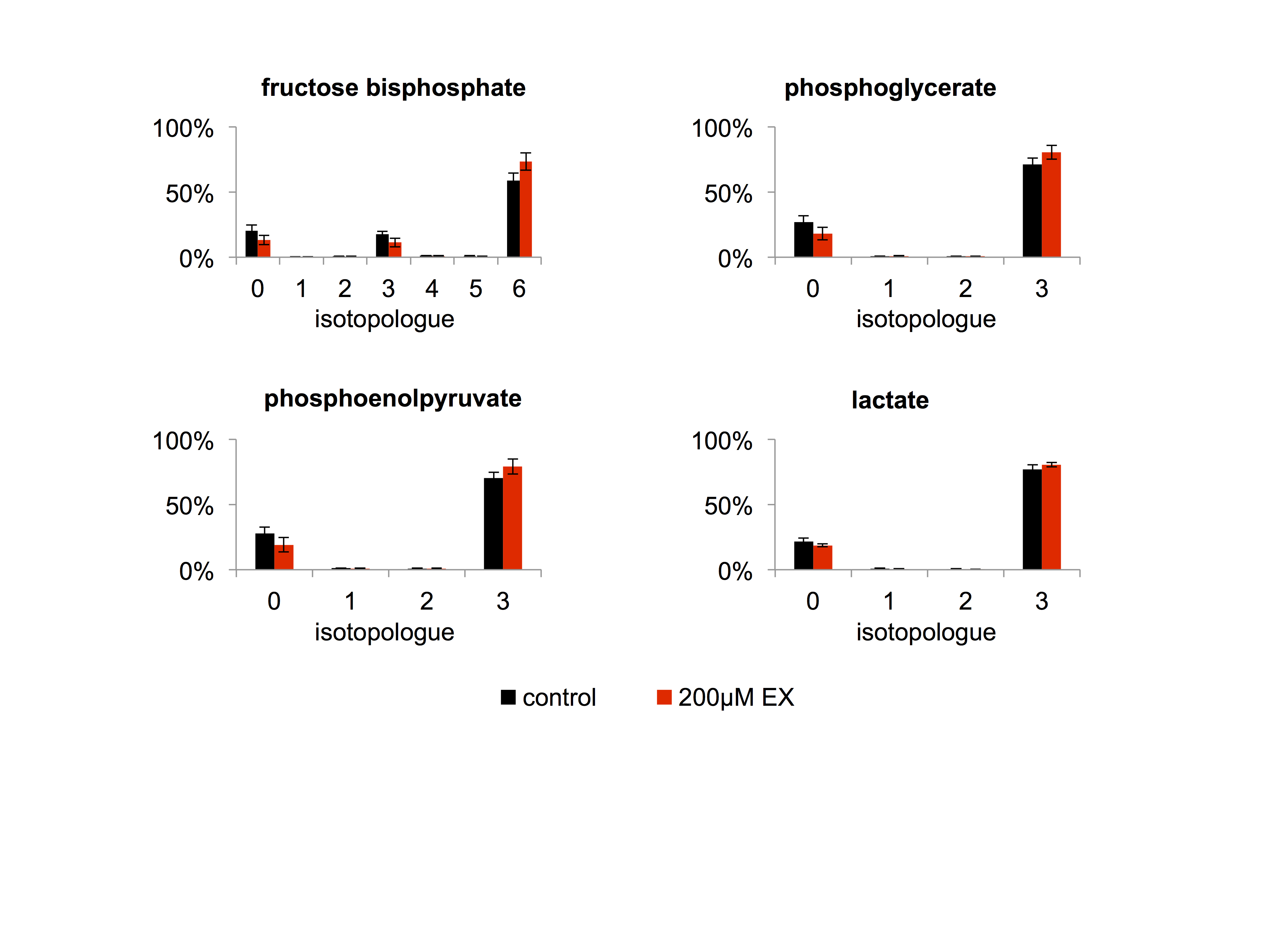

Supplement: S6 Fig — BT549 cells were treated with vehicle control or 200 μM etomoxir for 48 hours and then labeled with U-13C glucose for 12 hours in the presence of vehicle control or etomoxir (n = 3). Data are presented as mean ± SEM. (TIFF) [file pbio.2003782.s007.tiff]

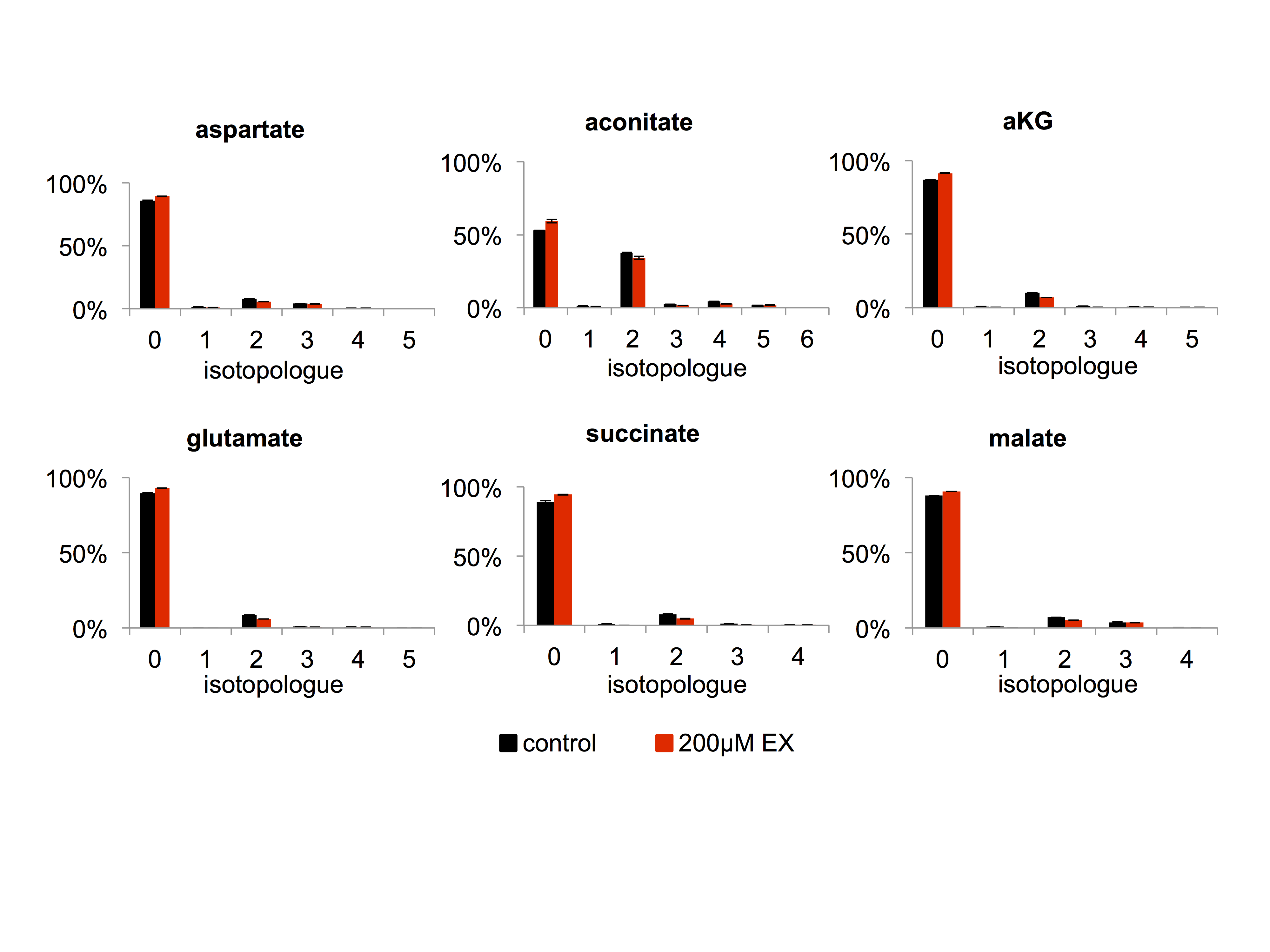

Supplement: S7 Fig — BT549 cells were treated with vehicle control or 200 μM etomoxir for 48 hours and then labeled with U-13C glucose for 12 hours in the presence of vehicle control or etomoxir (n = 3). Data are presented as mean ± SEM. (TIFF) [file pbio.2003782.s008.tiff]

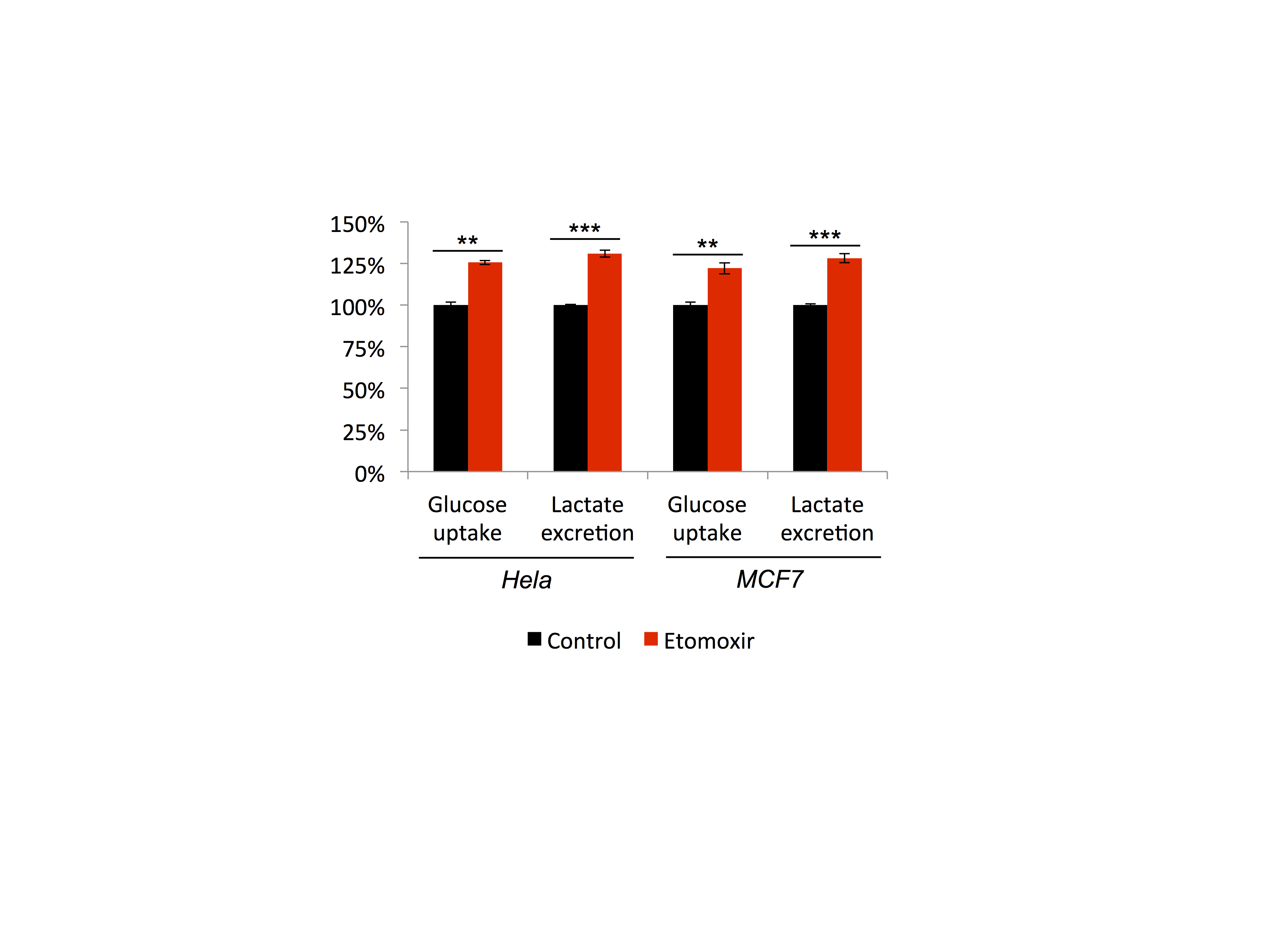

Supplement: S8 Fig — Data are presented as mean ± SEM. **p < 0.01, ***p < 0.001. (TIFF) [file pbio.2003782.s009.tiff]

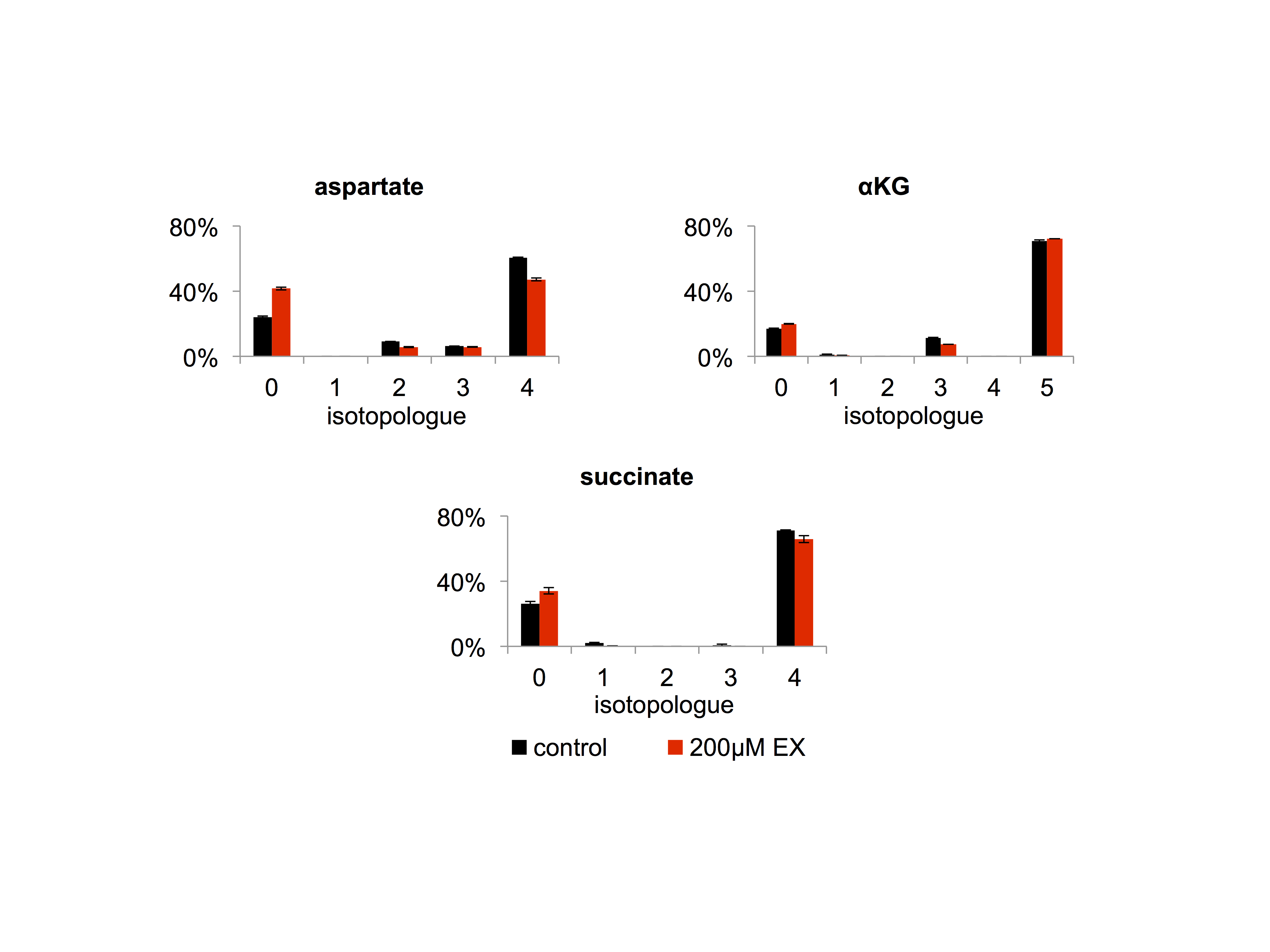

Supplement: S9 Fig — BT549 cells were treated with vehicle control or 200 μM etomoxir for 48 hours and then labeled with U-13C glutamine for 6 hours in the presence of vehicle control or etomoxir (n = 3). Data are presented as mean ± SEM. (TIFF) [file pbio.2003782.s010.tiff]

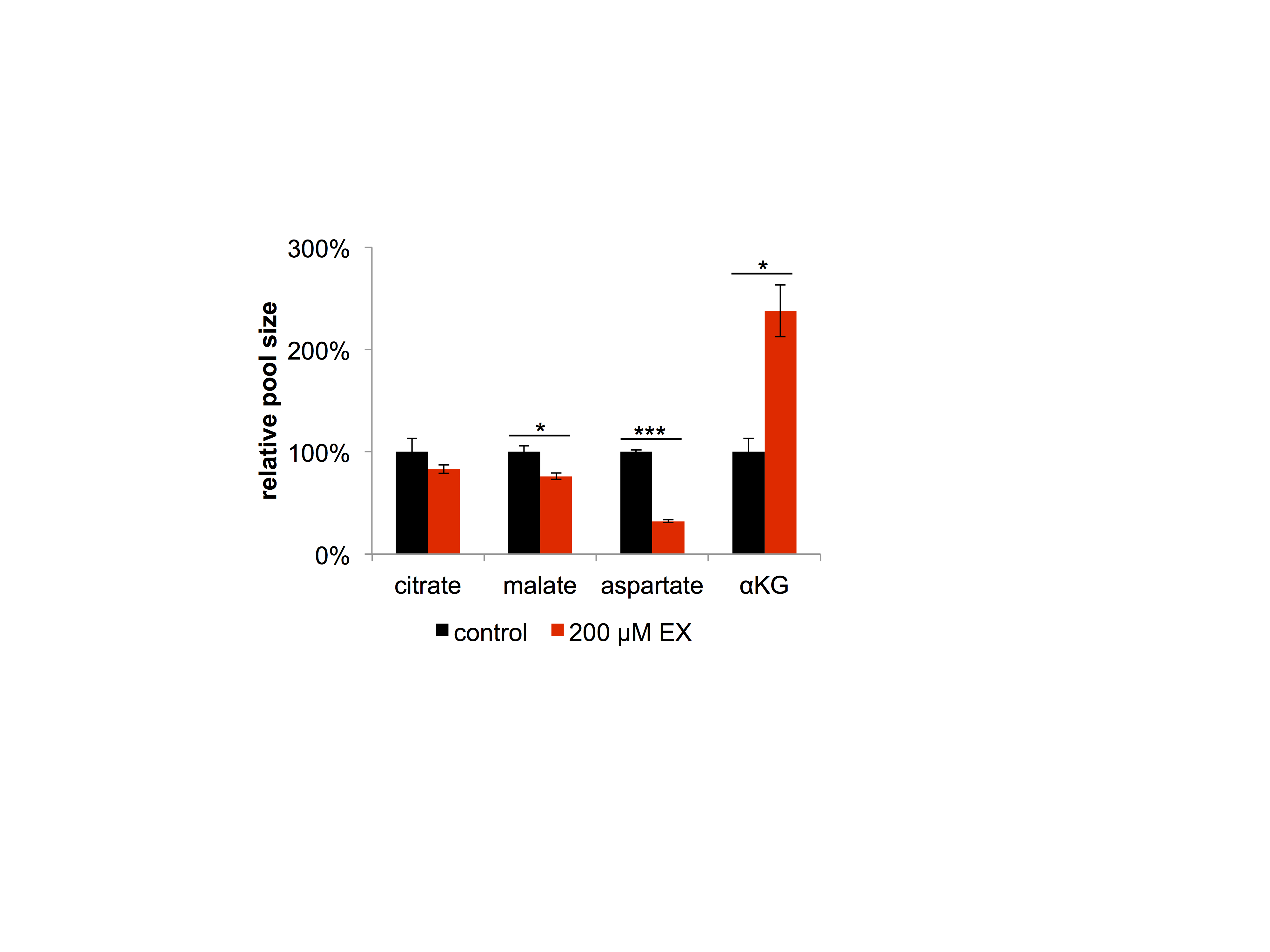

Supplement: S10 Fig — Pool sizes were normalized to cell dry mass, and deuterated phenylalanine (D8) was used as an internal standard. Data are presented as mean ± SEM. *p < 0.05, ***p < 0.001. (TIFF) [file pbio.2003782.s011.tiff]

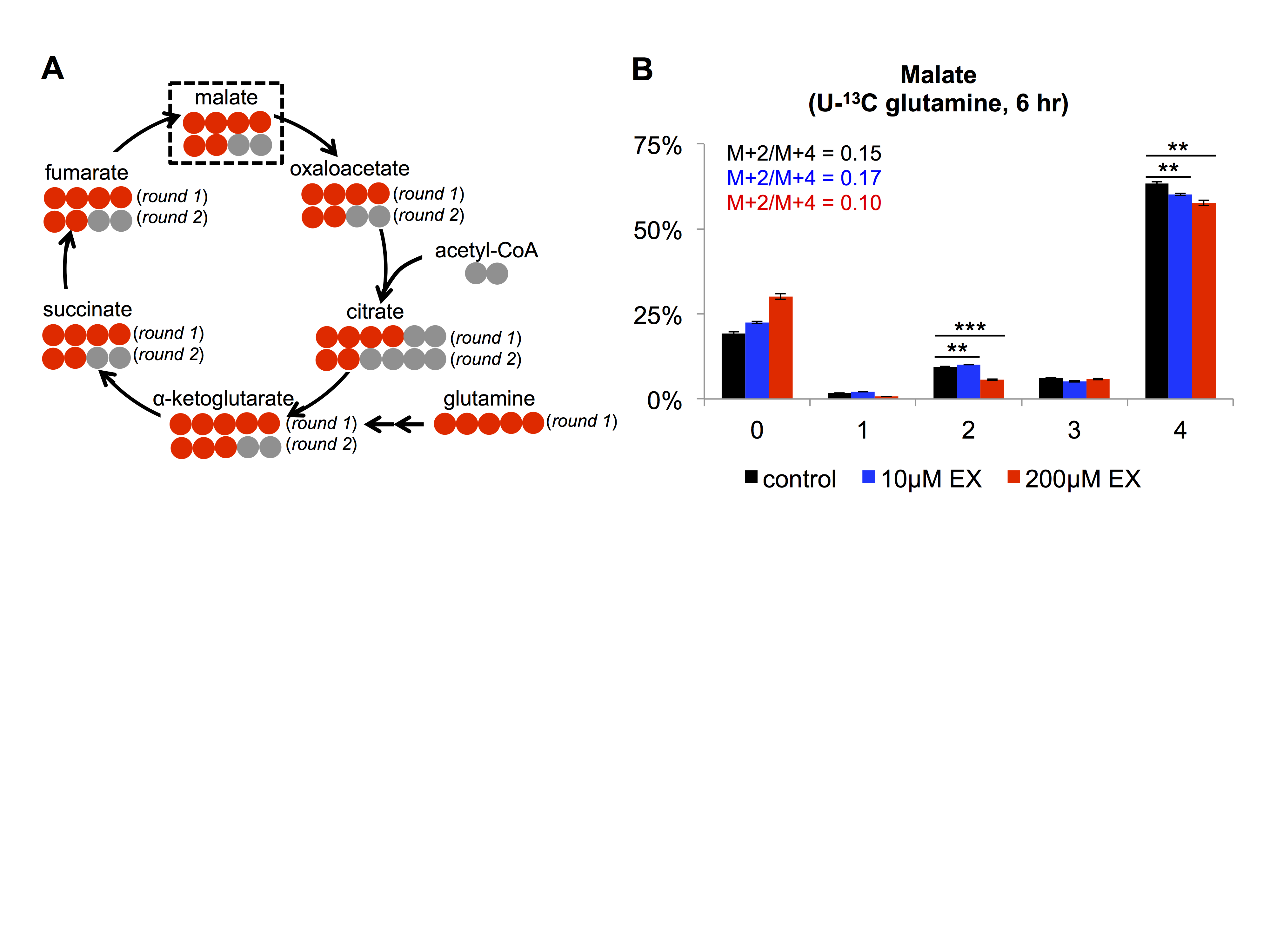

Supplement: S11 Fig — (A) Schematic showing the origin of the M+2 and M+4 isotopologues in the TCA cycle from U-13C glutamine. Red circles represent 13C-labeled carbon, and grey circles represent unlabeled carbon. (B) Isotopologue distribution pattern of malate after labeling with U-13C glutamine for 6 hours (n = 3). Data are presented as mean ± SEM. **p < 0.01, ***p < 0.001. (TIFF) [file pbio.2003782.s012.tiff]

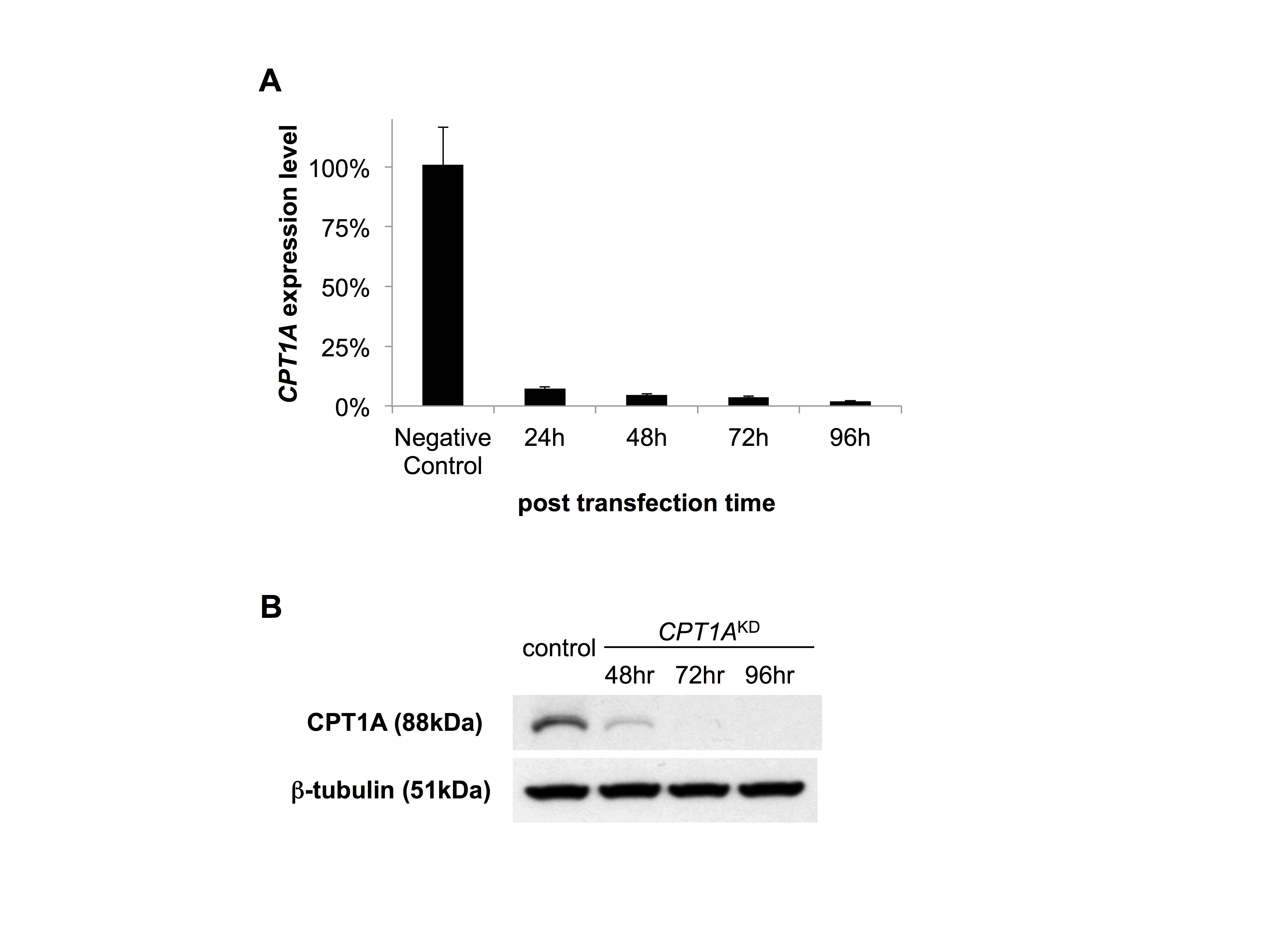

Supplement: S12 Fig — (A) CPT1A mRNA levels were determined by quantitative reverse transcription PCR (qRT-PCR) (normalized to an HPRT endogenous control) (n = 3). (B) Western blot analysis of cell lysate after siRNA knockdown for 48, 72, or 96 hours. β-tubulin was used as a loading control. Scrambled siRNA was used as negative control (control). (TIFF) [file pbio.2003782.s013.tiff]

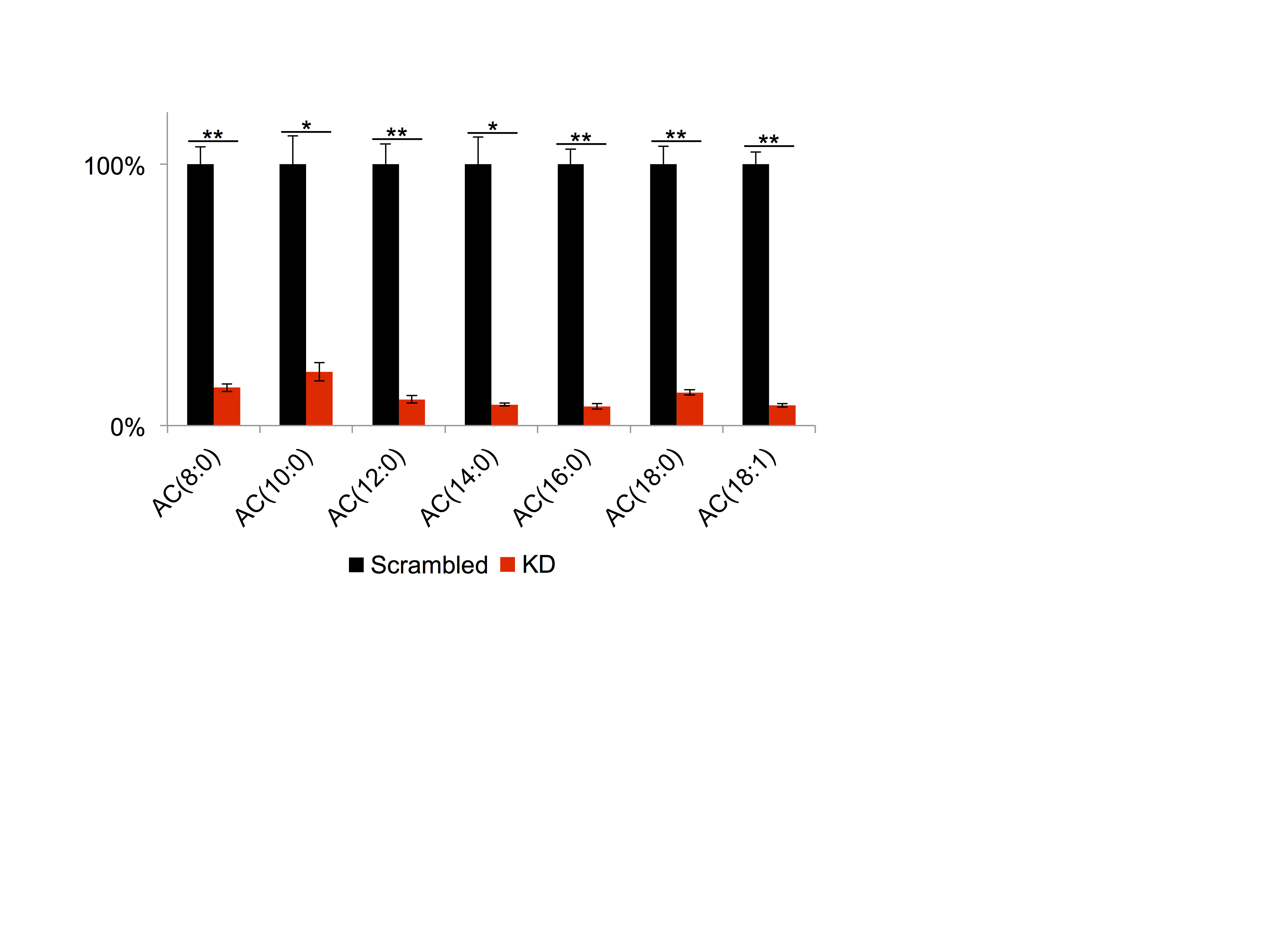

Supplement: S13 Fig — The acylcarnitine levels of long-chain fatty acids decreased by over 90%. Data are from cells harvested at 72 hours post small interfering RNA (siRNA) transfection (n = 3). Data are presented as mean ± SEM. *p < 0.05, **p < 0.01. (TIFF) [file pbio.2003782.s014.tiff]

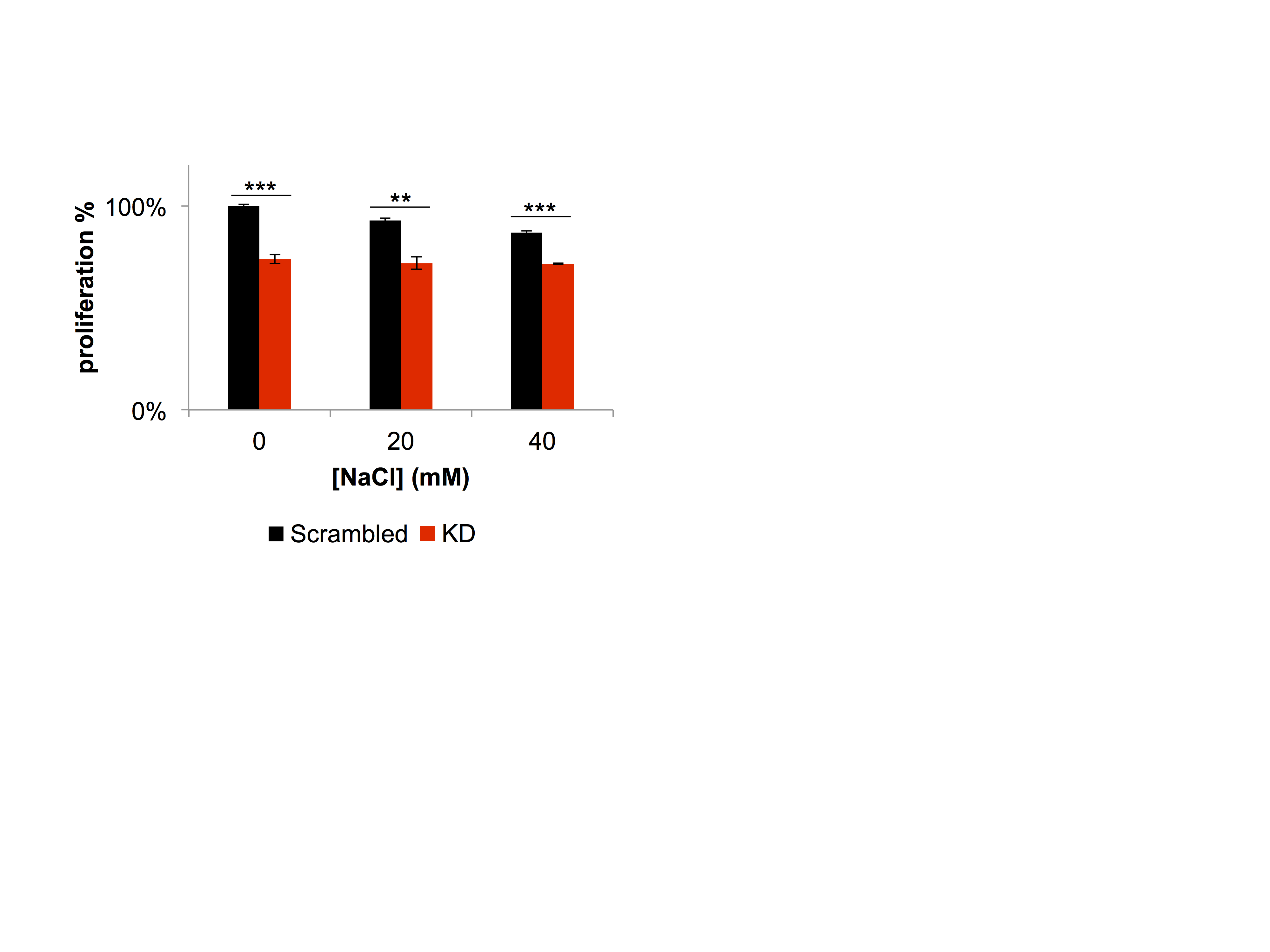

Supplement: S14 Fig — Data are presented as mean ± SEM. **p < 0.01, ***p < 0.001. (TIFF) [file pbio.2003782.s015.tiff]

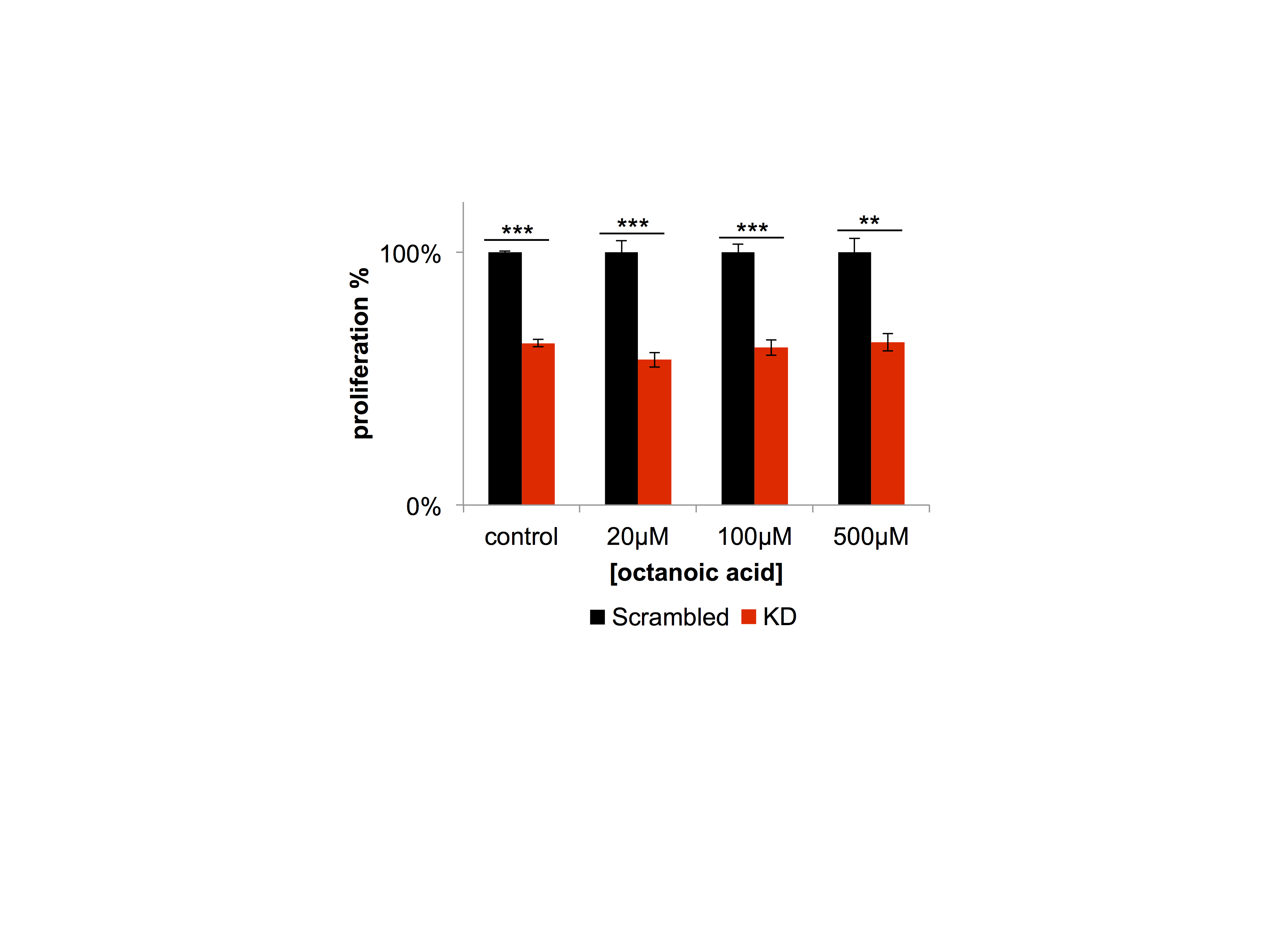

Supplement: S15 Fig — Data are presented as mean ± SEM. **p < 0.01, ***p < 0.001. (TIFF) [file pbio.2003782.s016.tiff]

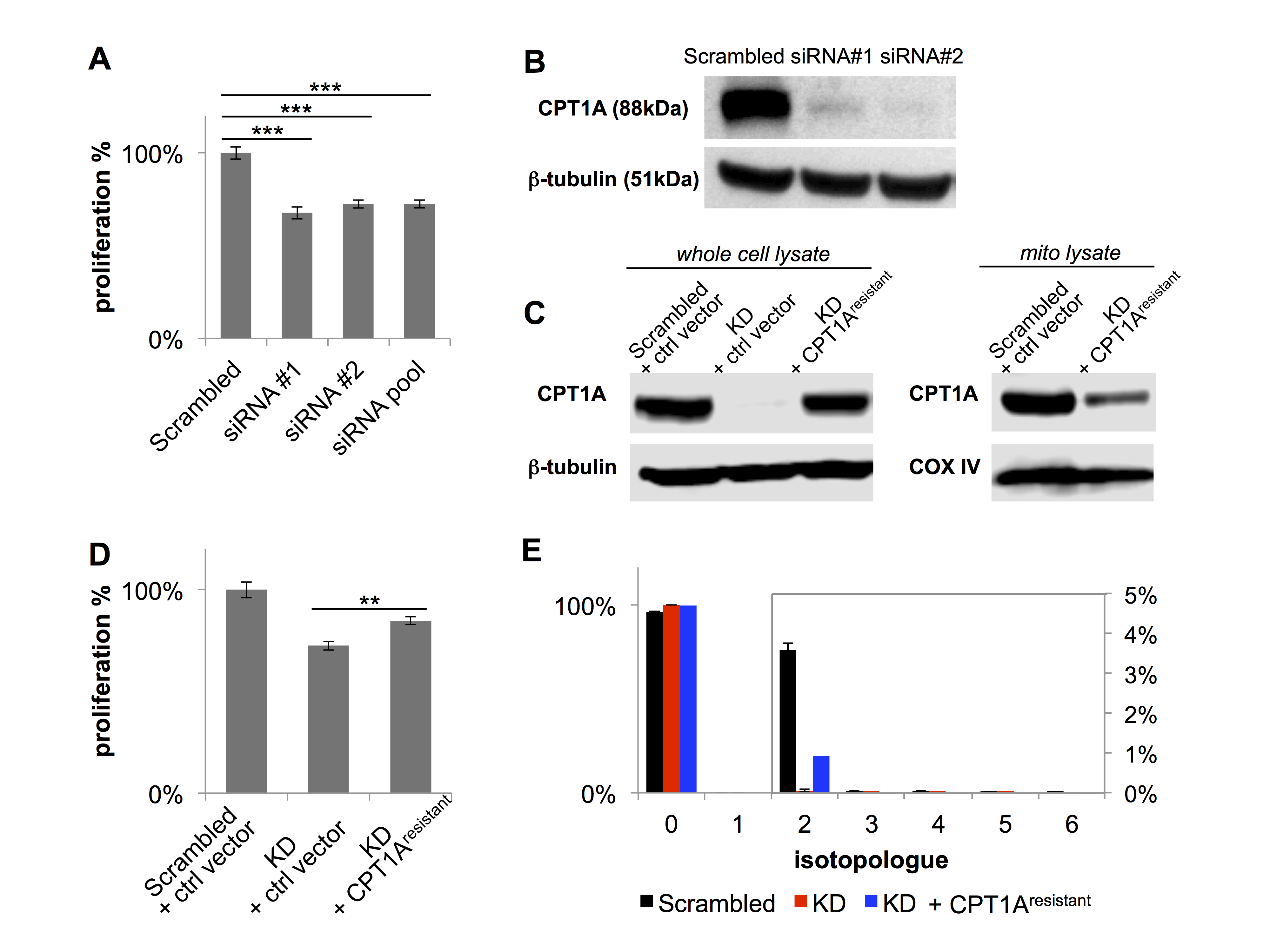

Supplement: S16 Fig — (A) Two different dicer-substrate short interfering RNA (DsiRNA) sequences (see S1 Text) were evaluated individually or as a pool (n = 5). They both resulted in a comparable decrease in BT549 cell proliferation. (B) Western blot analysis of cell lysates after small interfering RNA (siRNA) knockdown for 72 hours shows that both siRNA sequences resulted in decreased expression of CPT1A protein. (C) Western blot analysis of lysates from whole cells and isolated mitochondria shows that only some overexpressed CPT1A localized to mitochondria. (D) Overexpression of siRNA-resistant CPT1A (CPT1Aresistant) protein partially rescues the proliferation of CPT1AKD cells (n = 5). The DNA sequence for CPT1Aresistant is shown in S1 Text. The control vector was the same vector construct, but it expressed green fluorescent protein (GFP) instead of CPT1A. (E) Isotopologue distribution pattern of citrate after BT549 cells were labeled with 100 μM U-13C palmitate for 24 hours following a 72-hour knockdown and 48-hour overexpression. The M+2 isotopologue reflects fatty acid oxidation (FAO) activity. In CPT1AKD cells that overexpressed siRNA-resistant CPT1A, FAO activity was restored. All data are presented as mean ± SEM. **p < 0.01, ***p < 0.001. (TIFF) [file pbio.2003782.s017.tiff]

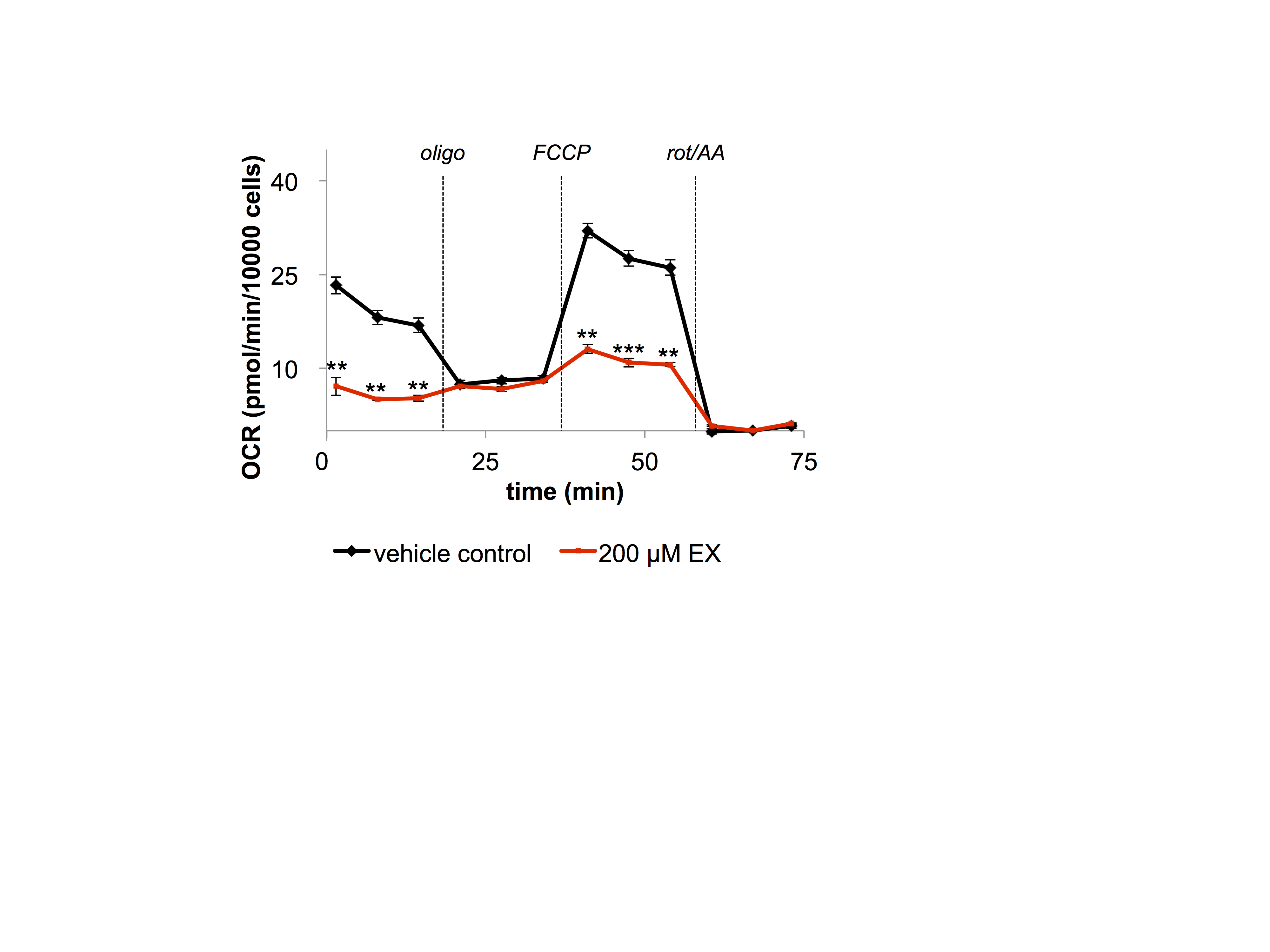

Supplement: S17 Fig — All data are presented as mean ± SEM. **p < 0.01, ***p < 0.001. The oxygen consumption rate (OCR) was corrected for nonmitochondrial respiration. (TIFF) [file pbio.2003782.s018.tiff]

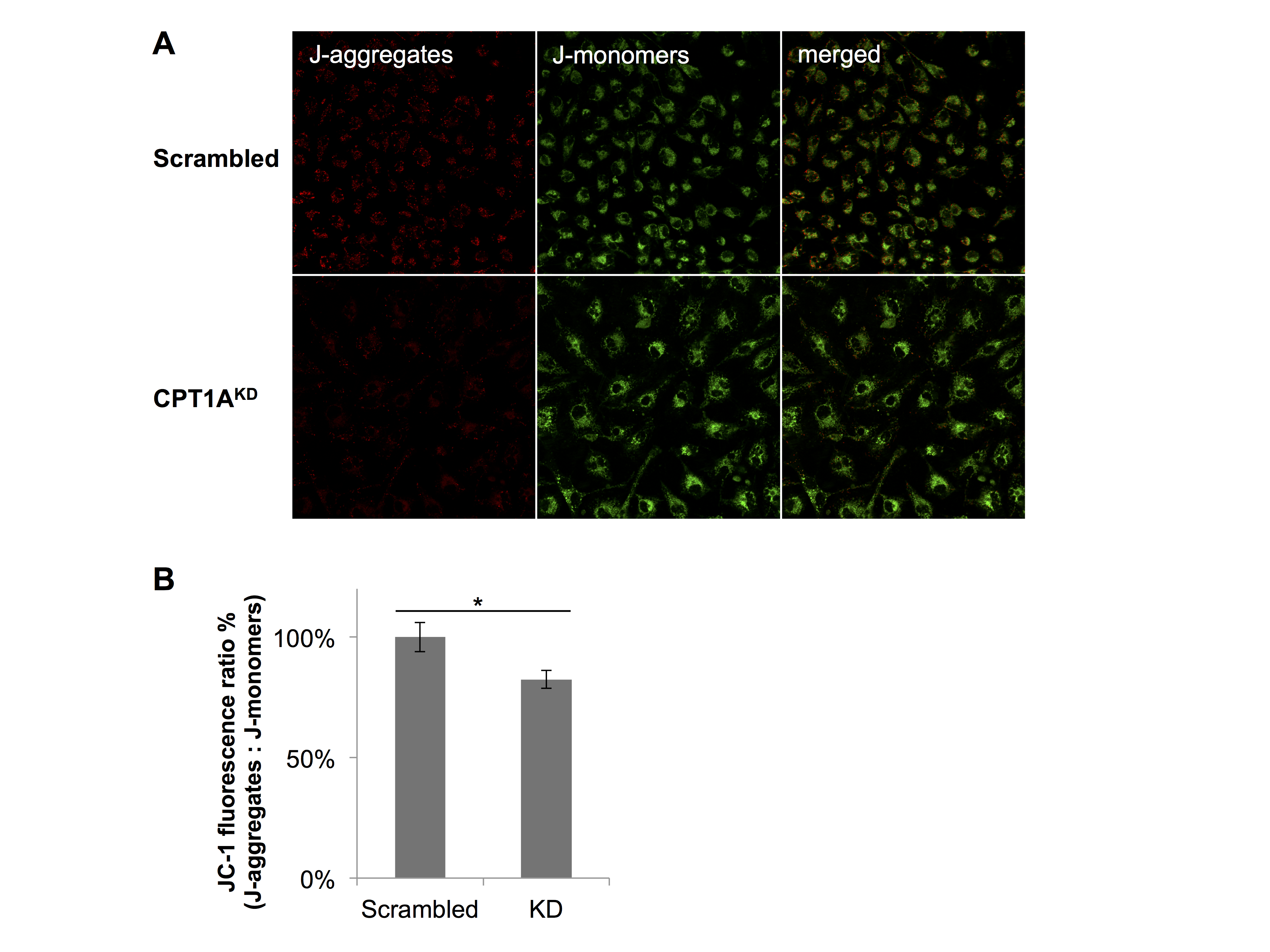

Supplement: S18 Fig — (A) After cells were treated with scrambled small interfering RNA (siRNA) or CPT1A siRNA for 72 hours, mitochondria were stained with JC-1. Red fluorescence of J-aggregates was detected by excitation with the 514-nm argon-ion laser source, and green fluorescence of J-monomers was detected with the 543-nm helium neon laser source. (B) The absolute fluorescence intensities of several representative images were quantified. The relative ratio of red J-aggregates to green J-monomers in scrambled siRNA controls and CPT1AKD cells was plotted (n = 5). Data are presented as mean ± SEM. *p < 0.05. (TIFF) [file pbio.2003782.s019.tiff]

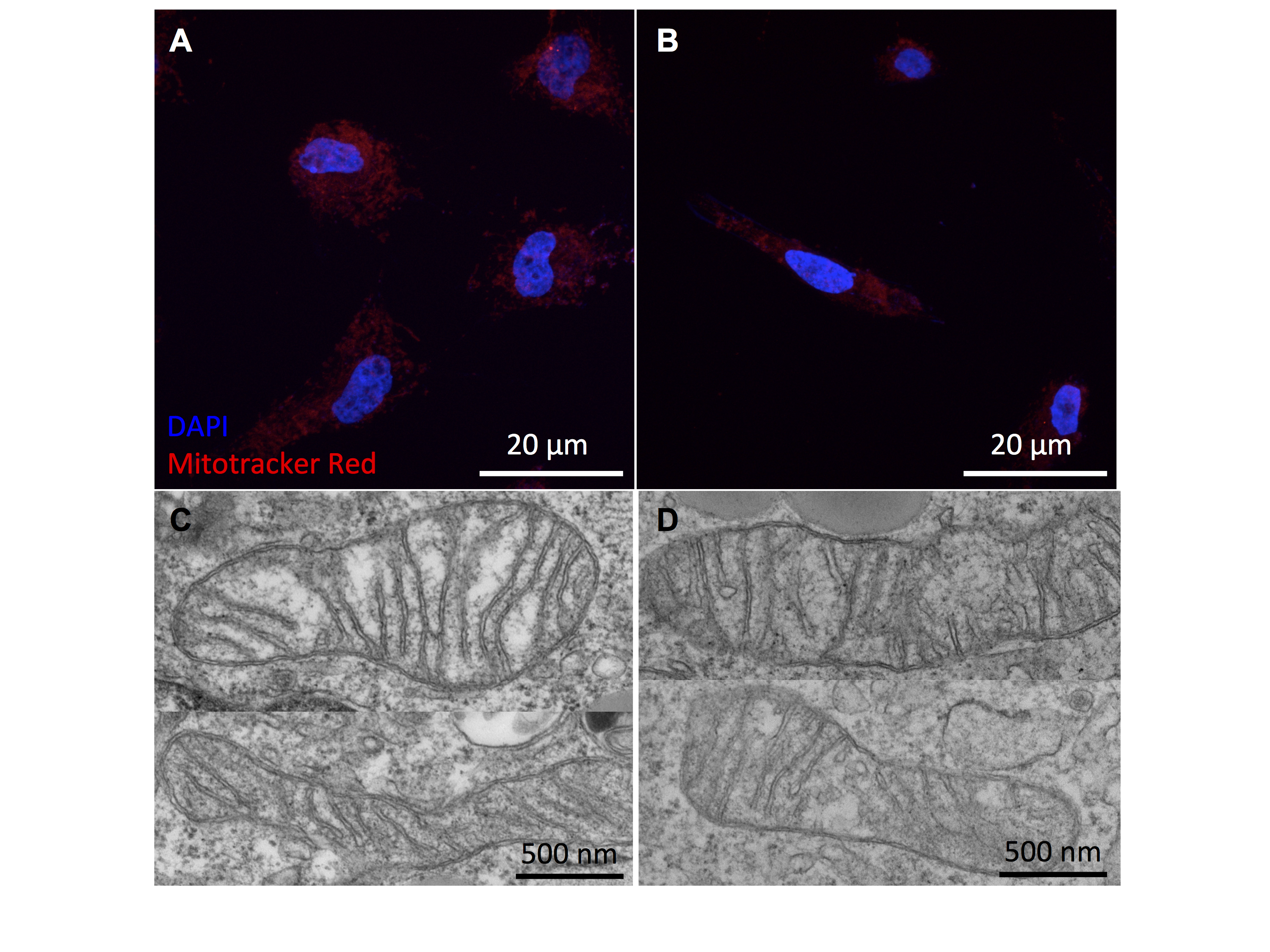

Supplement: S19 Fig — Etomoxir concentrations of (A) 10 μM and (B) 200 μM were tested. Mitochondria were stained by Mitotracker red, and nuclei were stained by DAPI. (C, D) Representative electron microscopy (EM) images of mitochondria from cells treated with (C) 10 μM etomoxir or (D) 200 μM etomoxir. (TIFF) [file pbio.2003782.s020.tiff]

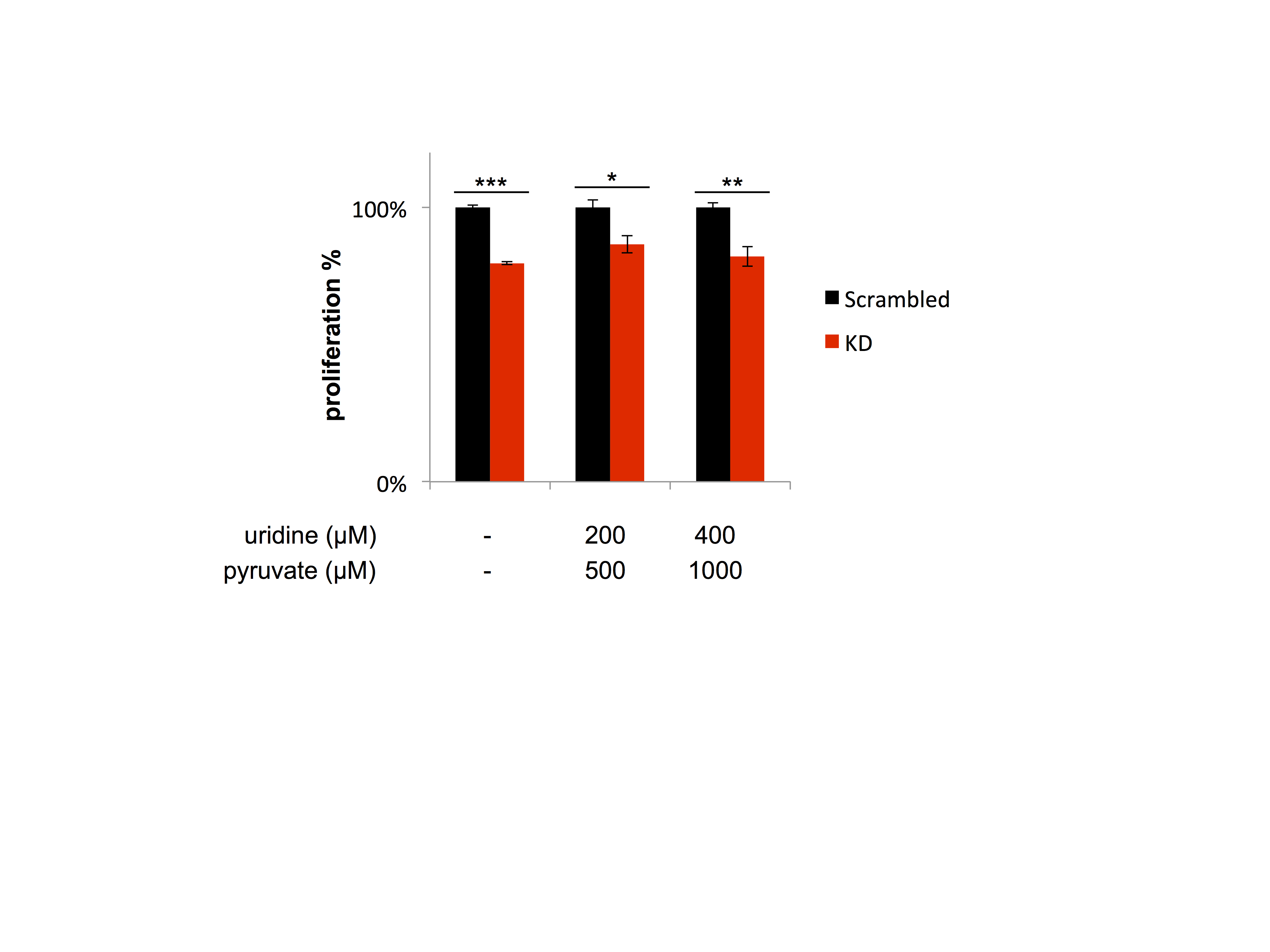

Supplement: S20 Fig — Data are presented as mean ± SEM. *p < 0.05, **p < 0.01, ***p < 0.001. (TIFF) [file pbio.2003782.s021.tiff]

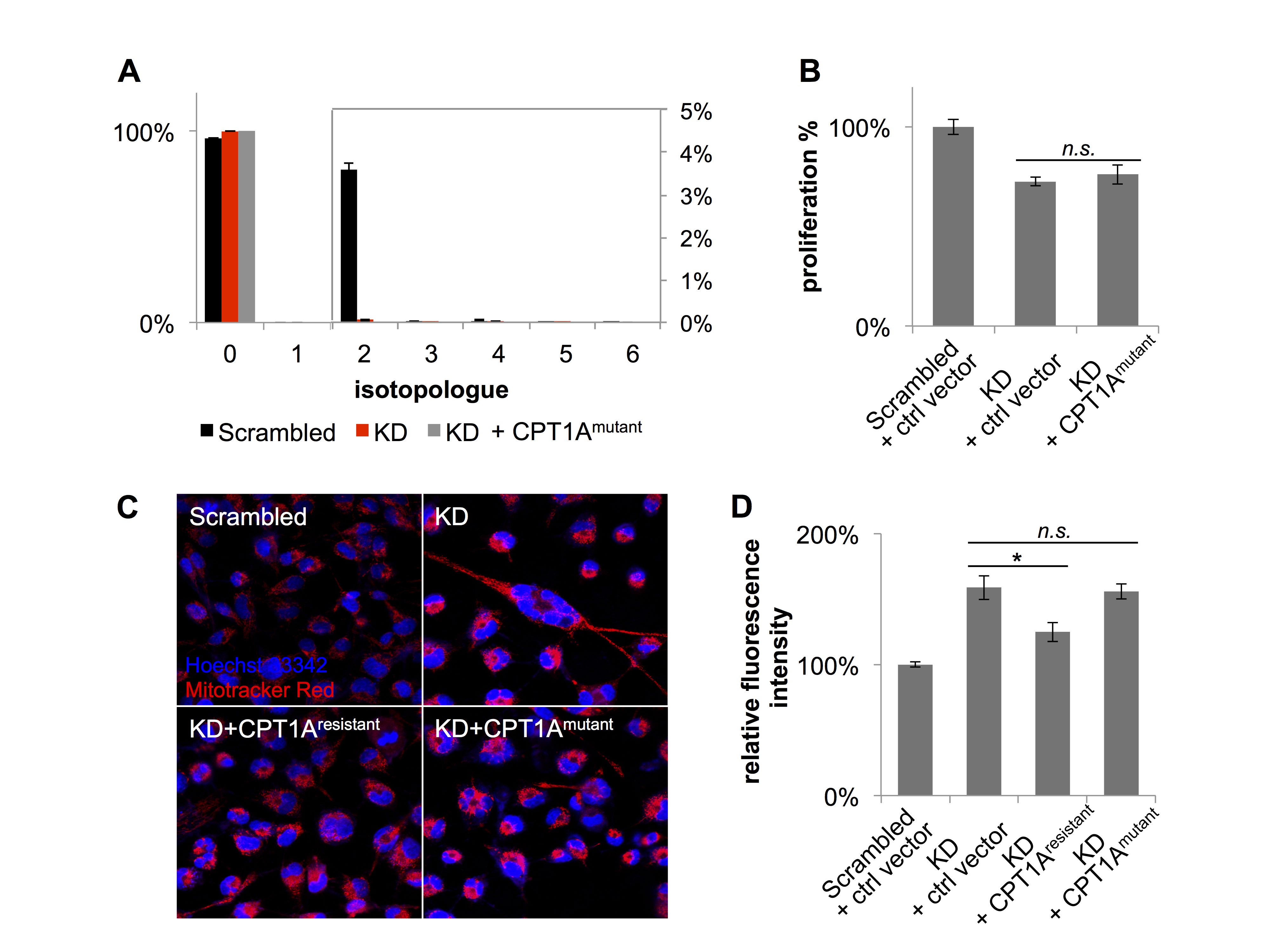

Supplement: S21 Fig — (A) The isotopologue distribution pattern of citrate after BT549 cells were labeled with 100 μM U-13C palmitate for 24 hours following a 72-hour knockdown and 48-hour overexpression. The M+2 isotopologue reflects fatty acid oxidation (FAO). As expected, in CPT1AKD cells that overexpressed a catalytically dead CPT1A, FAO was not restored. We note that the catalytically dead CPT1A protein is also resistant to knockdown by the small interfering RNA (siRNA) used. The control vector was the same as the vector construct, but it expressed green fluorescent protein (GFP) instead of CPT1A. (B) Overexpression of a catalytically dead CPT1A protein did not restore the proliferation of CPT1AKD cells (n = 5). (C) Mitochondria were stained by Mitotracker red, and nuclei were stained by Hoechst 33342. Quantitation of fluorescence intensity is shown in panel (D). (D) Total fluorescence intensity of Mitotracker red from 3 representative fields taken at 20× (n = 3). All data are presented as mean ± SEM. n.s., not statistically significant, *p < 0.05. (TIFF) [file pbio.2003782.s022.tiff]
