# Supplementary material for: Identifying off-target effects of etomoxir reveals that carnitine palmitoyltransferase I is essential for cancer cell proliferation independent of β-oxidation
Source: PLoS Biol. 2018 Mar 29;16(3):e2003782. doi: 10.1371/journal.pbio.2003782 (PMC5892939; doi:10.1371/journal.pbio.2003782)
Supplement: S1 Table — (DOCX) [file pbio.2003782.s023.docx]

**Table S1**. Dysregulated features identified by untargeted profiling.

| **Index** | **Fold change** | **p-value** | **Up/Down in *CTP1*^KD^** | **Accurate Mass (m/z)** | **Intensity in scrambled (ion counts)** | **Intensity in KD (ion counts)** |
| --- | --- | --- | --- | --- | --- | --- |
| 1 | 4.23 | 0.0004 | DOWN | 748.5897 | 1882438 | 445530 |
| 2 | 2.28 | 0.0018 | DOWN | 837.8149 | 321064 | 140514 |
| 3 | 2.28 | 0.0001 | DOWN | 836.8113 | 533668 | 234207 |
| 4 | 2.23 | 0.0001 | DOWN | 838.8174 | 100009 | 44849 |
| 5 | 2.20 | 0.0002 | DOWN | 1199.8169 | 96489 | 43922 |
| 6 | 2.12 | 0.0094 | DOWN | 698.5989 | 52344 | 24698 |
| 7 | 2.12 | 0.0023 | DOWN | 997.7408 | 348565 | 164586 |
| 8 | 2.12 | 0.0004 | DOWN | 954.7292 | 98471 | 46500 |
| 9 | 2.12 | 0.0015 | DOWN | 972.7402 | 365613 | 172763 |
| 10 | 2.10 | 0.0011 | DOWN | 996.7376 | 583179 | 277804 |
| 11 | 2.07 | 0.0057 | DOWN | 974.7555 | 343969 | 165883 |
| 12 | 2.07 | 0.0096 | DOWN | 697.5953 | 103703 | 50082 |
| 13 | 2.06 | 0.0095 | DOWN | 717.6321 | 46171 | 22370 |
| 14 | 2.06 | 0.0008 | DOWN | 973.7432 | 220788 | 107418 |
| 15 | 2.05 | 0.0068 | DOWN | 605.4579 | 270013 | 131937 |
| 16 | 2.03 | 0.0004 | DOWN | 838.8257 | 70135 | 34486 |
| 17 | 2.02 | 0.0032 | DOWN | 975.7588 | 210475 | 104450 |
| 18 | 2.01 | 0.0013 | DOWN | 998.7436 | 117873 | 58735 |
| 19 | 2.01 | 0.0033 | DOWN | 820.7867 | 45469 | 22675 |
| 20 | 1.99 | 0.0003 | DOWN | 1087.6902 | 261540 | 131497 |
| 21 | 1.98 | 0.0047 | DOWN | 1197.8014 | 106712 | 53986 |
| 22 | 1.96 | 0.0000 | DOWN | 1088.6933 | 165199 | 84391 |
| 23 | 1.96 | 0.0022 | DOWN | 819.7846 | 76257 | 38983 |
| 24 | 1.95 | 0.0056 | DOWN | 865.8457 | 288153 | 148128 |
| 25 | 1.94 | 0.0082 | DOWN | 946.7238 | 222076 | 114286 |
| 26 | 1.94 | 0.0014 | DOWN | 976.7619 | 72365 | 37372 |
| 27 | 1.92 | 0.0081 | DOWN | 622.6173 | 90757 | 47301 |
| 28 | 1.91 | 0.0098 | DOWN | 648.6328 | 216208 | 113017 |
| 29 | 1.91 | 0.0014 | DOWN | 693.5616 | 96498 | 50520 |
| 30 | 1.91 | 0.0051 | DOWN | 866.8485 | 91875 | 48155 |
| 31 | 1.89 | 0.0042 | DOWN | 864.8424 | 482606 | 255377 |
| 32 | 1.88 | 0.0030 | DOWN | 947.7271 | 129845 | 69020 |
| 33 | 1.88 | 0.0049 | DOWN | 847.8155 | 60071 | 31934 |
| 34 | 1.87 | 0.0047 | DOWN | 694.5649 | 48316 | 25813 |
| 35 | 1.85 | 0.0051 | DOWN | 1089.7060 | 66693 | 36016 |
| 36 | 1.83 | 0.0069 | DOWN | 786.6765 | 130580 | 71293 |
| 37 | 1.83 | 0.0022 | DOWN | 833.6723 | 1595427 | 871422 |
| 38 | 1.83 | 0.0002 | DOWN | 718.6541 | 478482 | 261806 |
| 39 | 1.82 | 0.0044 | DOWN | 577.5233 | 226008 | 123899 |
| 40 | 1.79 | 0.0025 | DOWN | 719.6574 | 248480 | 138495 |
| 41 | 1.78 | 0.0001 | DOWN | 720.6606 | 71220 | 40018 |
| 42 | 1.74 | 0.0099 | DOWN | 863.6321 | 227111 | 130211 |
| 43 | 1.72 | 0.0034 | DOWN | 834.7951 | 123978 | 72090 |
| 44 | 1.71 | 0.0014 | DOWN | 669.4417 | 114160 | 66713 |
| 45 | 1.71 | 0.0077 | DOWN | 851.7958 | 235974 | 137954 |
| 46 | 1.69 | 0.0068 | DOWN | 862.6291 | 427689 | 252536 |
| 47 | 1.68 | 0.0041 | DOWN | 844.6182 | 80107 | 47541 |
| 48 | 1.68 | 0.0088 | DOWN | 835.7987 | 71188 | 42383 |
| 49 | 1.68 | 0.0015 | DOWN | 905.6741 | 209906 | 125016 |
| 50 | 1.67 | 0.0012 | DOWN | 850.7925 | 395392 | 236996 |
| 51 | 1.66 | 0.0056 | DOWN | 565.5535 | 185893 | 111745 |
| 52 | 1.66 | 0.0024 | DOWN | 669.9434 | 84811 | 51084 |
| 53 | 1.65 | 0.0011 | DOWN | 890.6610 | 107666 | 65364 |
| 54 | 1.62 | 0.0021 | DOWN | 970.7218 | 118585 | 73329 |
| 55 | 1.57 | 0.0013 | DOWN | 721.6729 | 163376 | 104102 |
| 56 | 1.57 | 0.0071 | DOWN | 739.5584 | 161269 | 103042 |
| 57 | 1.56 | 0.0019 | DOWN | 720.6697 | 307162 | 196357 |
| 58 | 1.56 | 0.0081 | DOWN | 864.6449 | 173752 | 111514 |
| 59 | 1.56 | 0.0045 | DOWN | 865.6479 | 90675 | 58207 |
| 60 | 1.54 | 0.0001 | DOWN | 997.7405 | 81955 | 53155 |
| 61 | 1.54 | 0.0041 | DOWN | 729.6582 | 72712 | 47270 |
| 62 | 1.53 | 0.0009 | DOWN | 745.6730 | 154096 | 100890 |
| 63 | 1.53 | 0.0083 | DOWN | 746.6856 | 1232253 | 807549 |
| 64 | 1.52 | 0.0029 | DOWN | 728.6081 | 345612 | 226832 |
| 65 | 1.51 | 0.0028 | DOWN | 729.6115 | 163476 | 108244 |
| 66 | 1.51 | 0.0028 | DOWN | 710.5974 | 223603 | 148303 |
| 67 | 1.50 | 0.0098 | DOWN | 747.6888 | 682059 | 453350 |
| 68 | 1.54 | 0.0029 | UP | 329.2689 | 53418 | 82524 |
| 69 | 1.56 | 0.0070 | UP | 825.5864 | 107045 | 167419 |
| 70 | 1.69 | 0.0034 | UP | 843.7352 | 121126 | 204969 |
| 71 | 1.86 | 0.0004 | UP | 760.4993 | 35747 | 66651 |
| 72 | 2.20 | 0.0008 | UP | 545.3453 | 39319 | 86476 |
| 73 | 2.37 | 0.0007 | UP | 544.3420 | 114401 | 271627 |
| 74 | 3.53 | 0.0085 | UP | 290.2701 | 25293 | 89346 |
| 75 | 3.66 | 0.0003 | UP | 747.5777 | 341128 | 1250058 |
| 76 | 7.82 | 0.0017 | UP | 805.5629 | 8437 | 65954 |
| 77 | 8.48 | 0.0026 | UP | 565.5700 | 7000 | 59326 |
