# Supplementary material for: Identifying off-target effects of etomoxir reveals that carnitine palmitoyltransferase I is essential for cancer cell proliferation independent of β-oxidation
Source: PLoS Biol. 2018 Mar 29;16(3):e2003782. doi: 10.1371/journal.pbio.2003782 (PMC5892939; doi:10.1371/journal.pbio.2003782)
Supplement: S1 Text — (DOCX) [file pbio.2003782.s024.docx]

**Appendix S1.** Sequence for DsiRNA, CPT1A^resistant^ and CPT1A^mutant^

**The DsiRNA sequence for siRNA #1:**

5’- GCCUUUACGUGGUGUCUAAAUAUCT-3’

3’- GACGGAAAUGCACCACAGAUUUAUAGA-5’

**The DsiRNA sequence for siRNA #2:**

5’- UCAAUGGACAGCUACGCCAAAUCTC-3’

3’- GCAGUUACCUGUCGAUGCGGUUUAGAG-5’

**The DNA sequence for CPT1A^resistant^:**

atggcagaagctcaccaagctgtggcctttcagttcacggtcactccggacgggattgacctgcggctgagccacgaggccttgcggcagatttatctctctggacttcattcctggaaaaagaagttcatcagattcaagaacggcatcatcactggcgtgtacccggcaagcccctccagttggcttatcgtggtggtgggcgtgatgacaacgatgtacgccaagatcgacccctcgttaggaataattgcaaaaatcaatcggactctggaaacggccaactgcatgtccagccagacgaagaacgtggtcagcggcgtgctgtttggcaccggcctgtgggtggccctcatcgtcaccatgcgctactccctgaaagtgctgctctcctaccacgggtggatgttcactgagcacggcaagatgagtcgtgccaccaagatctggatgggtatggtcaagatcttttcaggccgaaaacccatgttgtacagcttccagacatcgctgcctcgcctgccggtcccggctgtcaaagacactgtgaacaggtatctacagtcggtgaggcctcttatgaaggaagaagacttcaaacggatgacagcacttgctcaagattttgctgtcggtcttggaccaagattacagtggtatttgaagttaaaatcctggtgggctacaaattacgtgagcgactggtgggaggagtacatctacctccgaggacgagggccgctcatggtgaacagcaactattatgccatggatctgctgtatatccttccaactcacattcaggcagcaagagccggcaacgccatccatgccatcctgctttacaggcgcaaactggaccgggaggaaatcaaaccaattcgtcttttgggatccacgattccactctgctccgctcagtgggagcggatgtttaatacttcccggatcccaggagaggagacagacaccatccagcacatgagagacagcaagcacatcgtcgtgtaccatcgaggacgctacttcaaggtctggctctaccatgatgggcggctgctgaagccccgggagatggagcagcagatgcagaggatcctggacaatacctcggagcctcagcccggggaggccaggctggcagccctcaccgcaggagacagagttccctgggccaggtgtcgtcaggcctattttggacgtgggaaaaataagcagtctcttgatgctgtggagaaagcagcgttcttcgtgacgttagatgaaactgaagaaggatacagaagtgaagacccggataccagcatggattcttatgctaaatctctactacacggccgatgttacgacaggtggtttgacaagtcgttcacgtttgttgtcttcaaaaacgggaagatgggcctcaacgctgaacactcctgggcagatgcgccgatcgtggcccacctttgggagtacgtcatgtccattgacagcctccagctgggctatgcggaggatgggcactgcaaaggcgacatcaatccgaacattccgtaccccaccaggctgcagtgggacatcccgggggaatgtcaagaggttatagagacctccctgaacaccgcaaatcttctggcaaacgacgtggatttccattccttcccattcgtagcctttggtaaaggaatcatcaagaaatgtcgcacgagcccagacgcctttgtgcagctggccctccagctggcgcactacaaggacatgggcaagttttgcctcacatacgaggcctccatgacccggctcttccgagaggggaggacggagaccgtgcgctcctgcaccactgagtcatgcgacttcgtgcgggccatggtggacccggcccagacggtggaacagaggctgaagttgttcaagttggcgtctgagaagcatcagcatatgtatcgcctcgccatgaccggctctgggatcgatcgtcacctcttttgtttgtatgtcgtcagcaaatatctcgctgtggagtcccctttccttaaggaagttttatctgagccttggagattatcaacaagccagacccctcagcagcaagtggagctgtttgacttggagaataacccagagtacgtgtccagcggagggggctttggaccggttgctgatgacggctatggtgtgtcgtacatccttgtgggagagaacctcatcaatttccacatttcttccaagttctcttgccctgagacggattctcatcgctttggaaggcacctgaaagaagcaatgactgacatcatcactttgtttggtctcagttctaattccaaaaagtaa

**The DNA sequence for CPT1A^mutant^ (G709E, G710E):**

atggcagaagctcaccaagctgtggcctttcagttcacggtcactccggacgggattgacctgcggctgagccacgaggccttgcggcagatttatctctctggacttcattcctggaaaaagaagttcatcagattcaagaacggcatcatcactggcgtgtacccggcaagcccctccagttggcttatcgtggtggtgggcgtgatgacaacgatgtacgccaagatcgacccctcgttaggaataattgcaaaaatcaatcggactctggaaacggccaactgcatgtccagccagacgaagaacgtggtcagcggcgtgctgtttggcaccggcctgtgggtggccctcatcgtcaccatgcgctactccctgaaagtgctgctctcctaccacgggtggatgttcactgagcacggcaagatgagtcgtgccaccaagatctggatgggtatggtcaagatcttttcaggccgaaaacccatgttgtacagcttccagacatcgctgcctcgcctgccggtcccggctgtcaaagacactgtgaacaggtatctacagtcggtgaggcctcttatgaaggaagaagacttcaaacggatgacagcacttgctcaagattttgctgtcggtcttggaccaagattacagtggtatttgaagttaaaatcctggtgggctacaaattacgtgagcgactggtgggaggagtacatctacctccgaggacgagggccgctcatggtgaacagcaactattatgccatggatctgctgtatatccttccaactcacattcaggcagcaagagccggcaacgccatccatgccatcctgctttacaggcgcaaactggaccgggaggaaatcaaaccaattcgtcttttgggatccacgattccactctgctccgctcagtgggagcggatgtttaatacttcccggatcccaggagaggagacagacaccatccagcacatgagagacagcaagcacatcgtcgtgtaccatcgaggacgctacttcaaggtctggctctaccatgatgggcggctgctgaagccccgggagatggagcagcagatgcagaggatcctggacaatacctcggagcctcagcccggggaggccaggctggcagccctcaccgcaggagacagagttccctgggccaggtgtcgtcaggcctattttggacgtgggaaaaataagcagtctcttgatgctgtggagaaagcagcgttcttcgtgacgttagatgaaactgaagaaggatacagaagtgaagacccggataccagcatggattcttatgctaaatctctactacacggccgatgttacgacaggtggtttgacaagtcgttcacgtttgttgtcttcaaaaacgggaagatgggcctcaacgctgaacactcctgggcagatgcgccgatcgtggcccacctttgggagtacgtcatgtccattgacagcctccagctgggctatgcggaggatgggcactgcaaaggcgacatcaatccgaacattccgtaccccaccaggctgcagtgggacatcccgggggaatgtcaagaggttatagagacctccctgaacaccgcaaatcttctggcaaacgacgtggatttccattccttcccattcgtagcctttggtaaaggaatcatcaagaaatgtcgcacgagcccagacgcctttgtgcagctggccctccagctggcgcactacaaggacatgggcaagttttgcctcacatacgaggcctccatgacccggctcttccgagaggggaggacggagaccgtgcgctcctgcaccactgagtcatgcgacttcgtgcgggccatggtggacccggcccagacggtggaacagaggctgaagttgttcaagttggcgtctgagaagcatcagcatatgtatcgcctcgccatgaccggctctgggatcgatcgtcacctcttttgtttgtatgtcgtcagcaaatatctcgctgtggagtcccctttccttaaggaagttttatctgagccttggagattatcaacaagccagacccctcagcagcaagtggagctgtttgacttggagaataacccagagtacgtgtccagcgaggagggctttggaccggttgctgatgacggctatggtgtgtcgtacatccttgtgggagagaacctcatcaatttccacatttcttccaagttctcttgccctgagacggattctcatcgctttggaaggcacctgaaagaagcaatgactgacatcatcactttgtttggtctcagttctaattccaaaaagtaa
